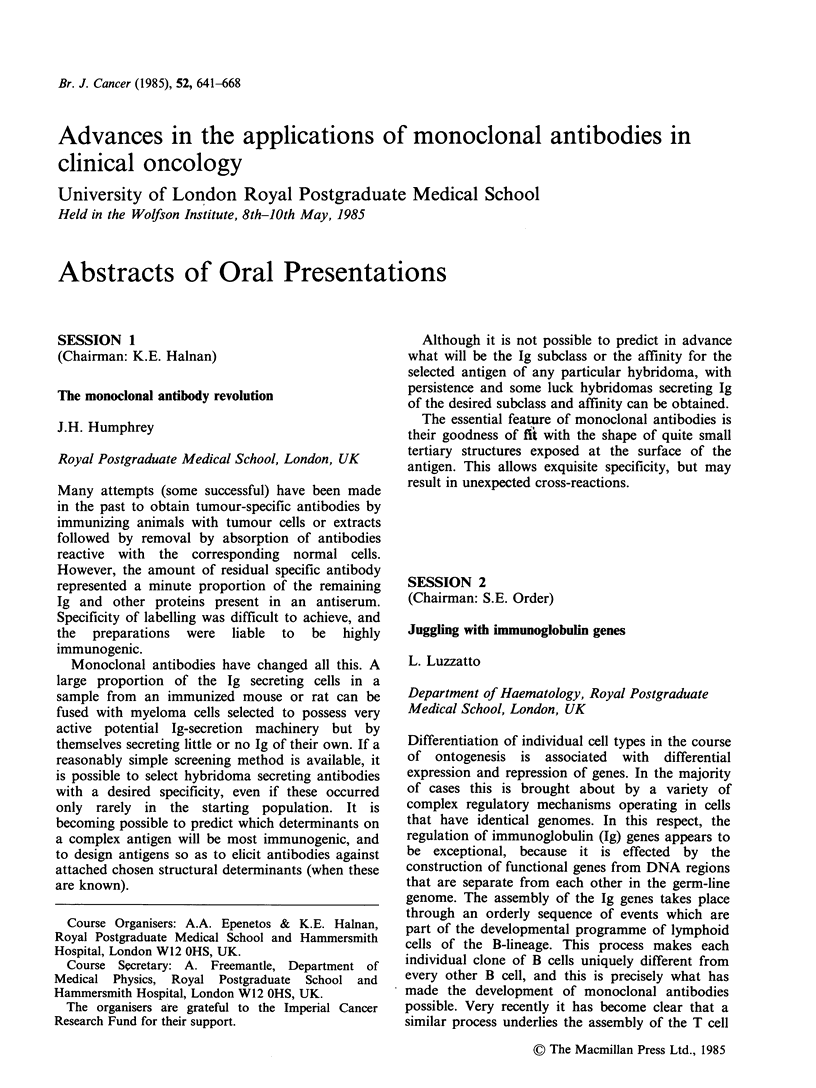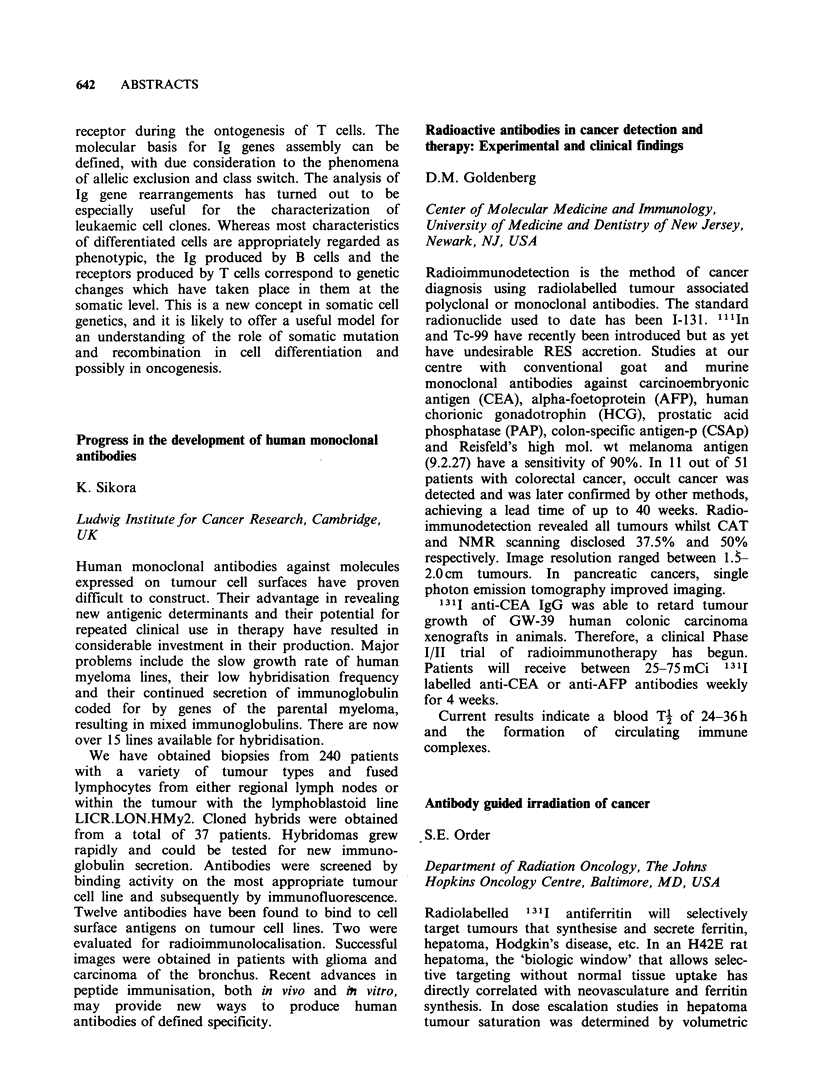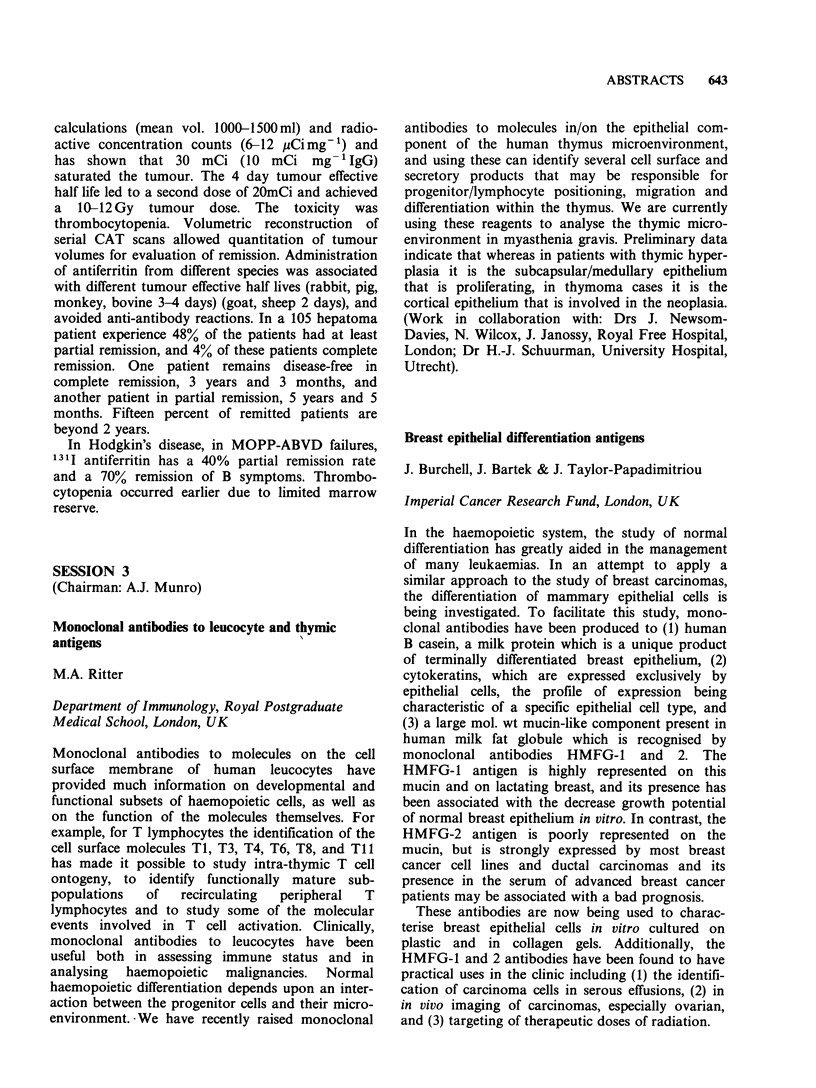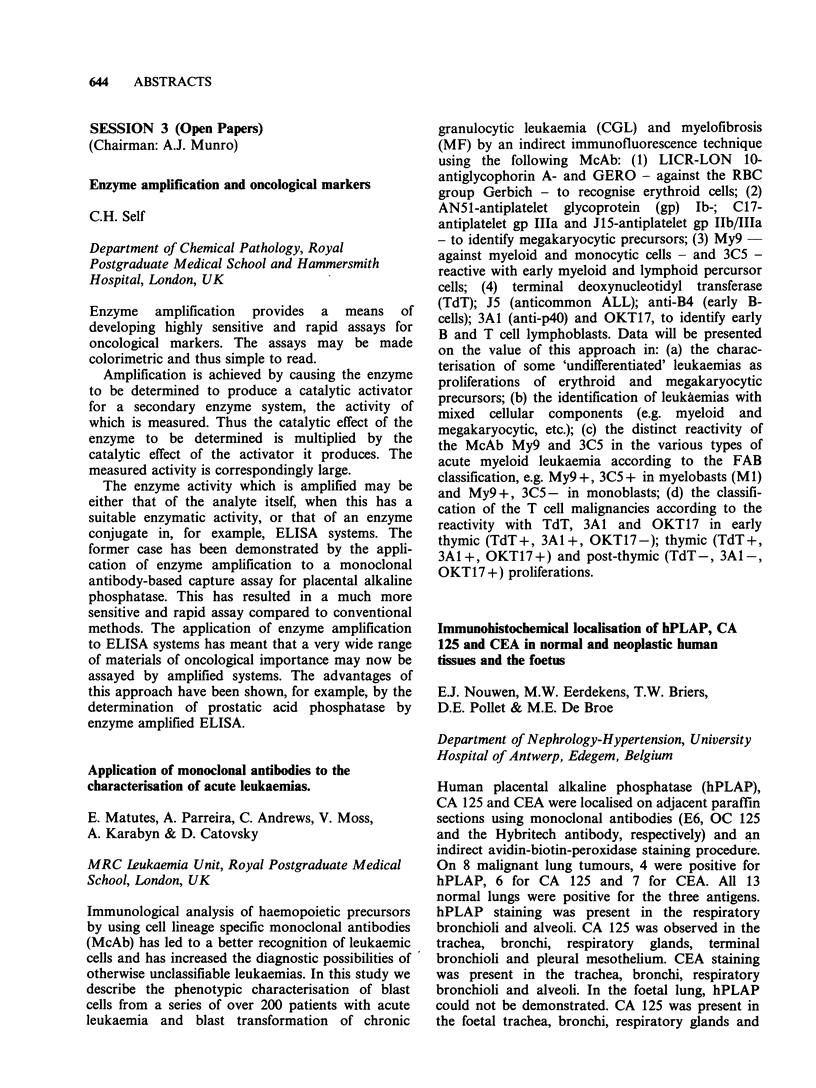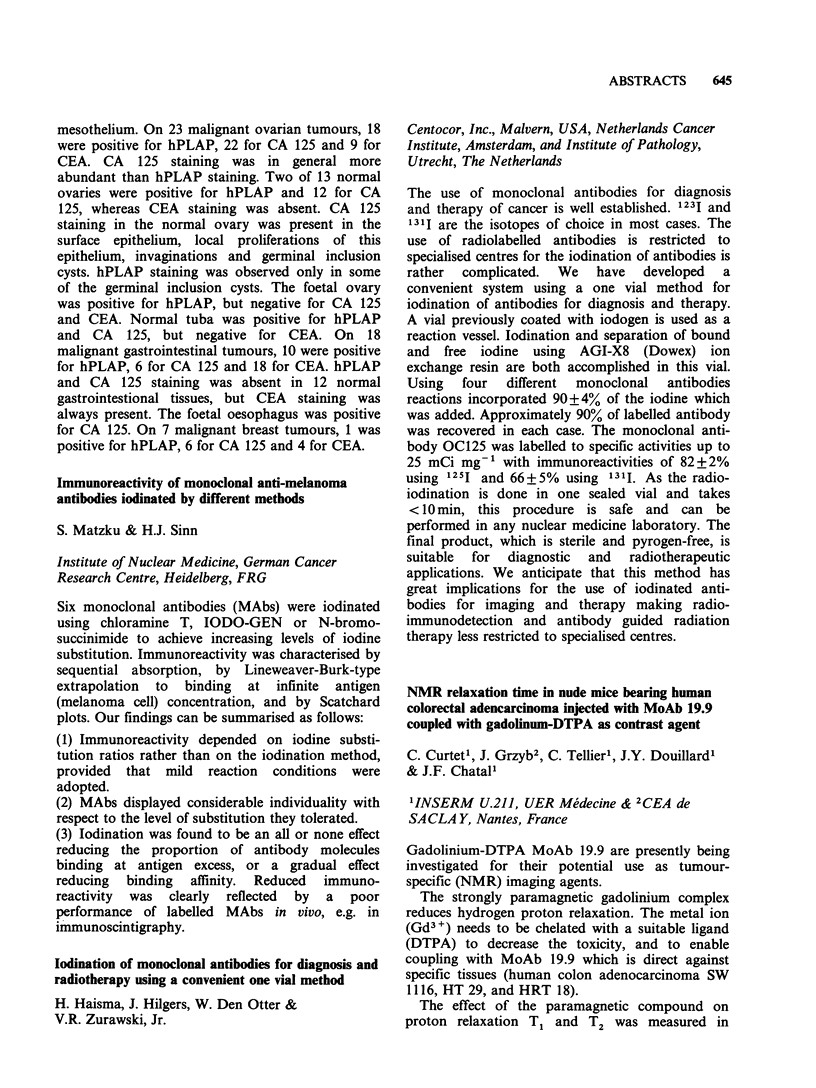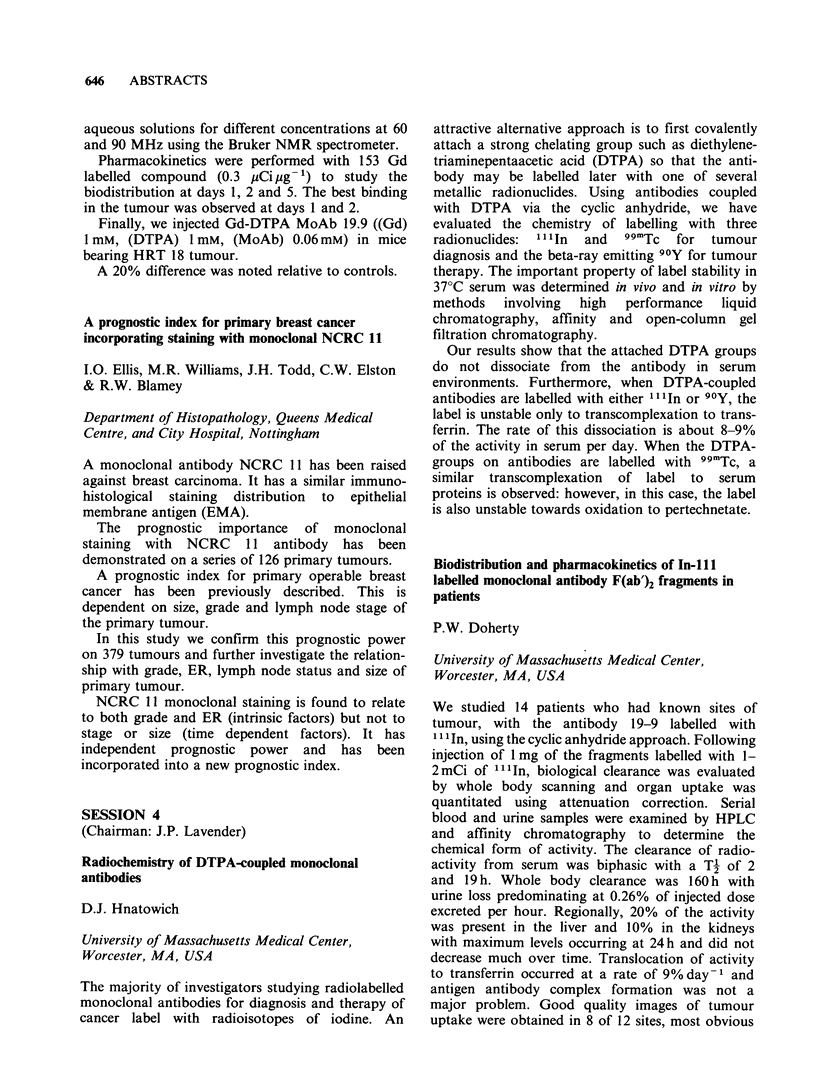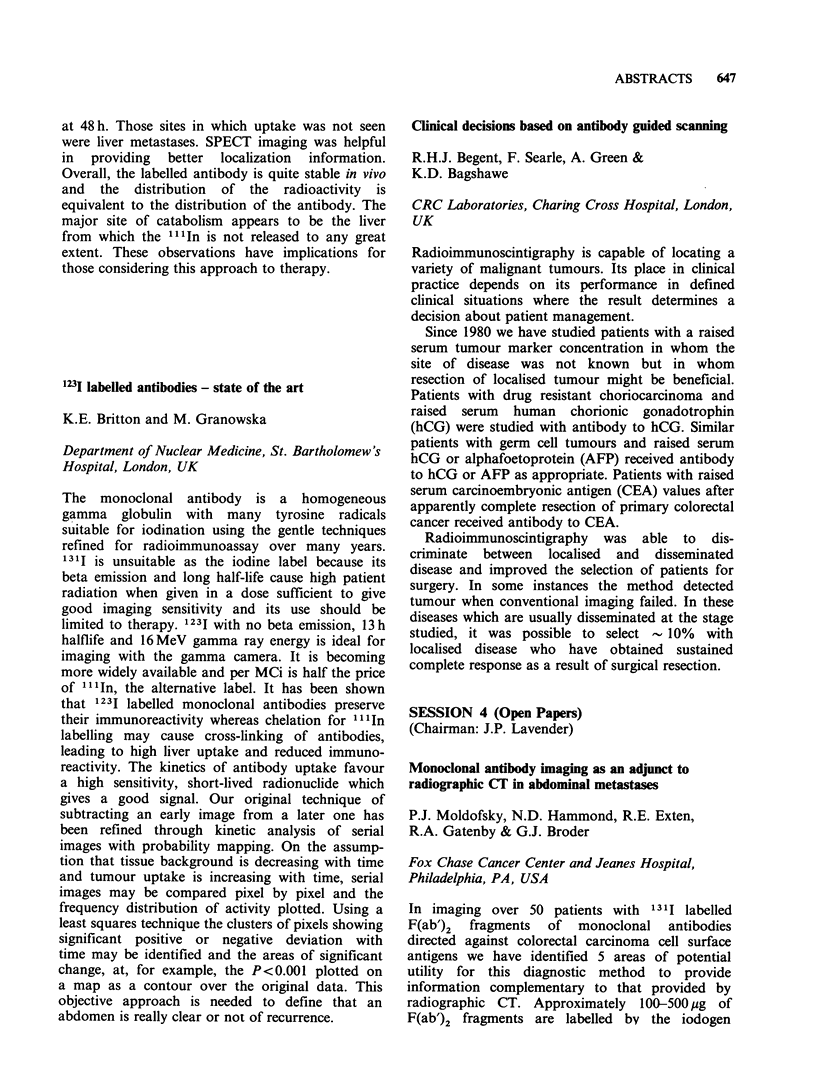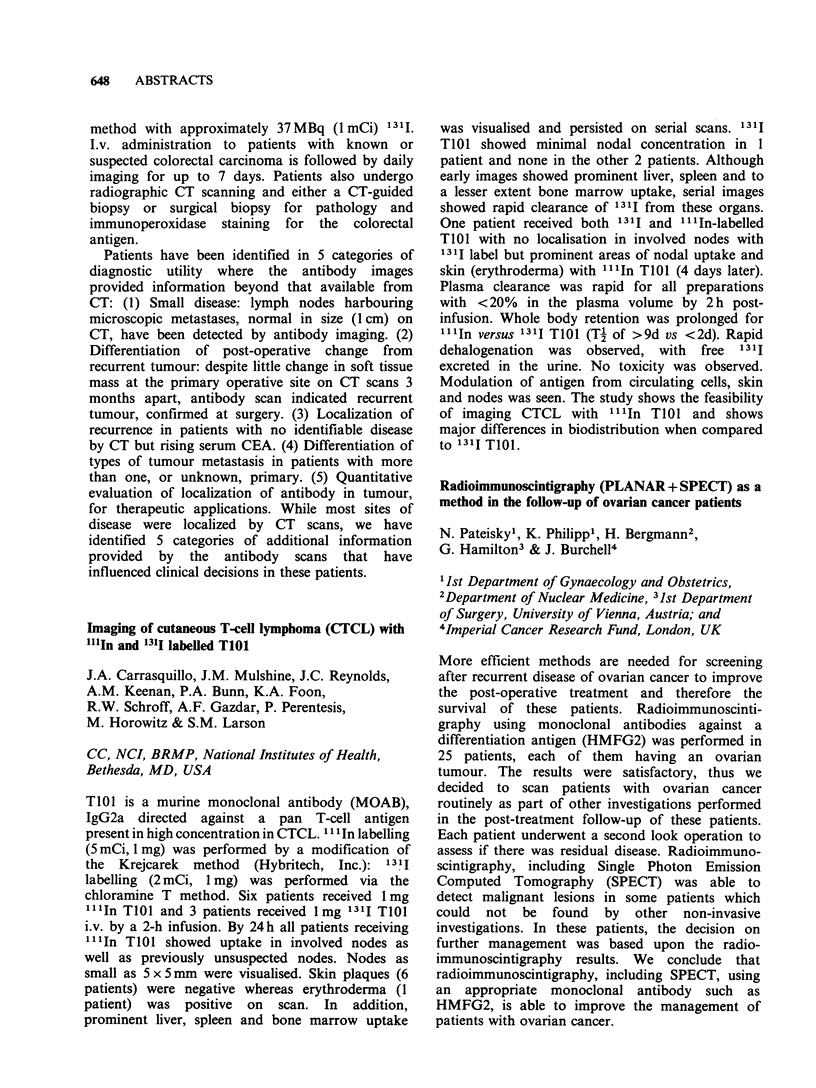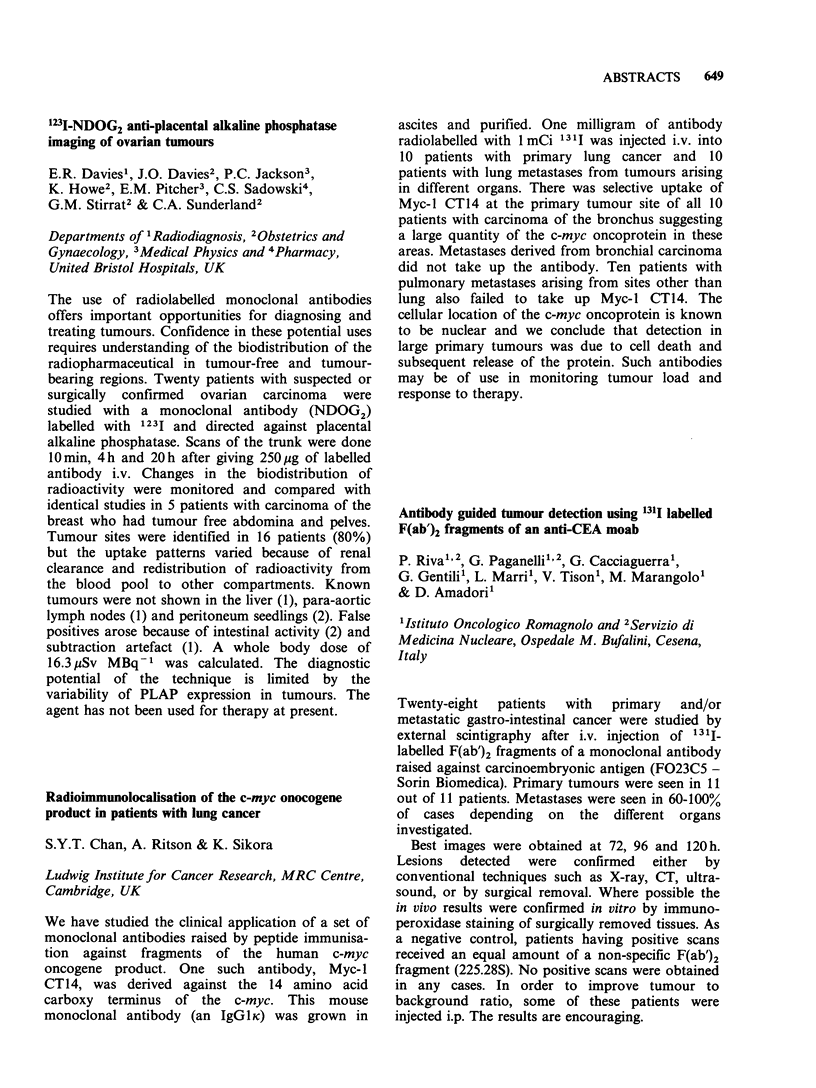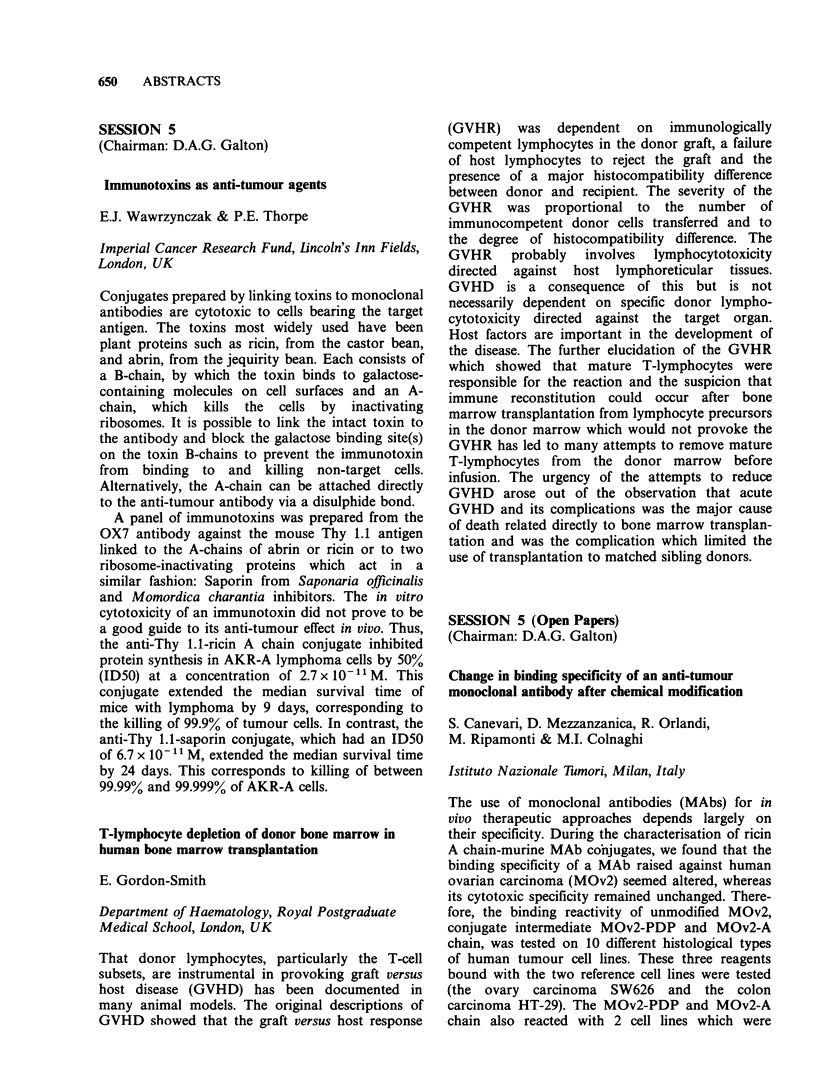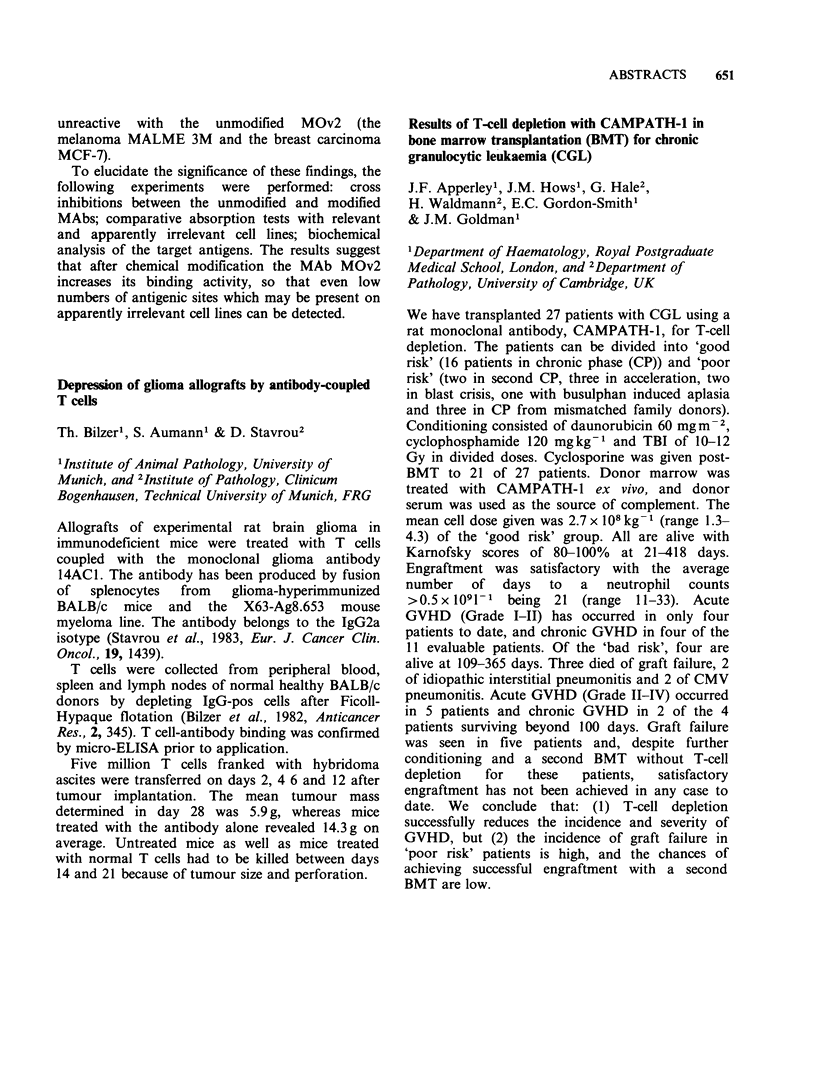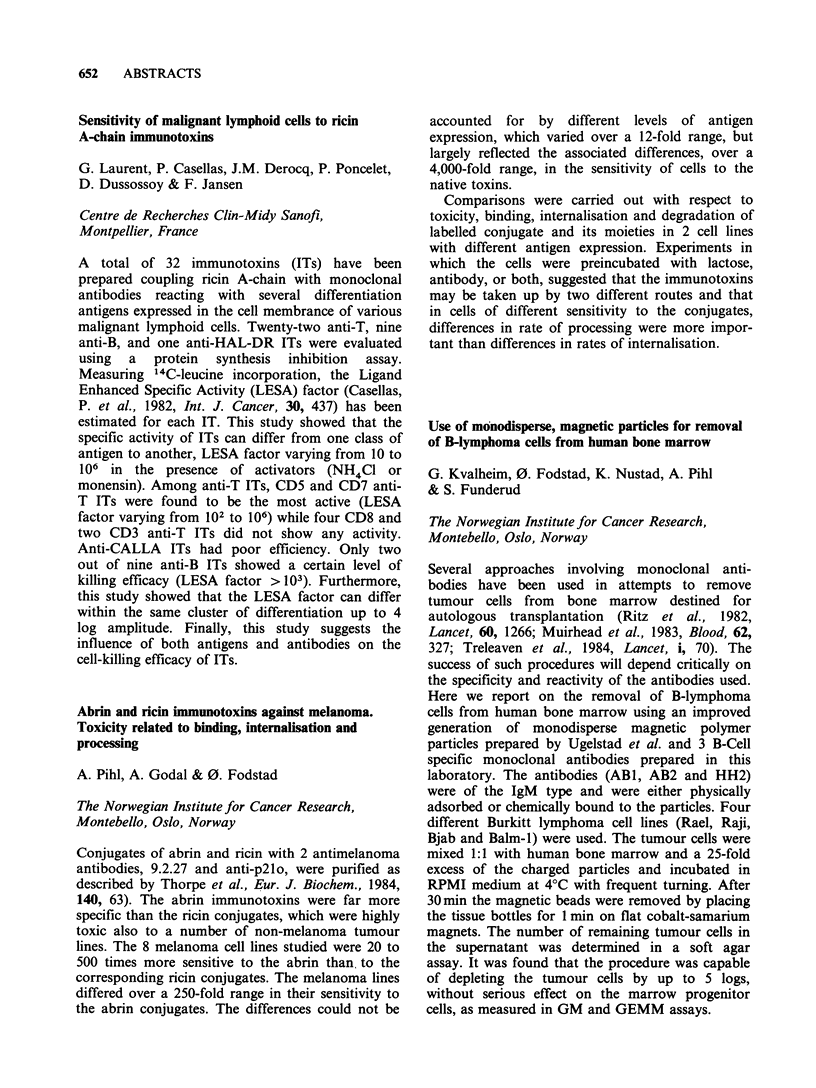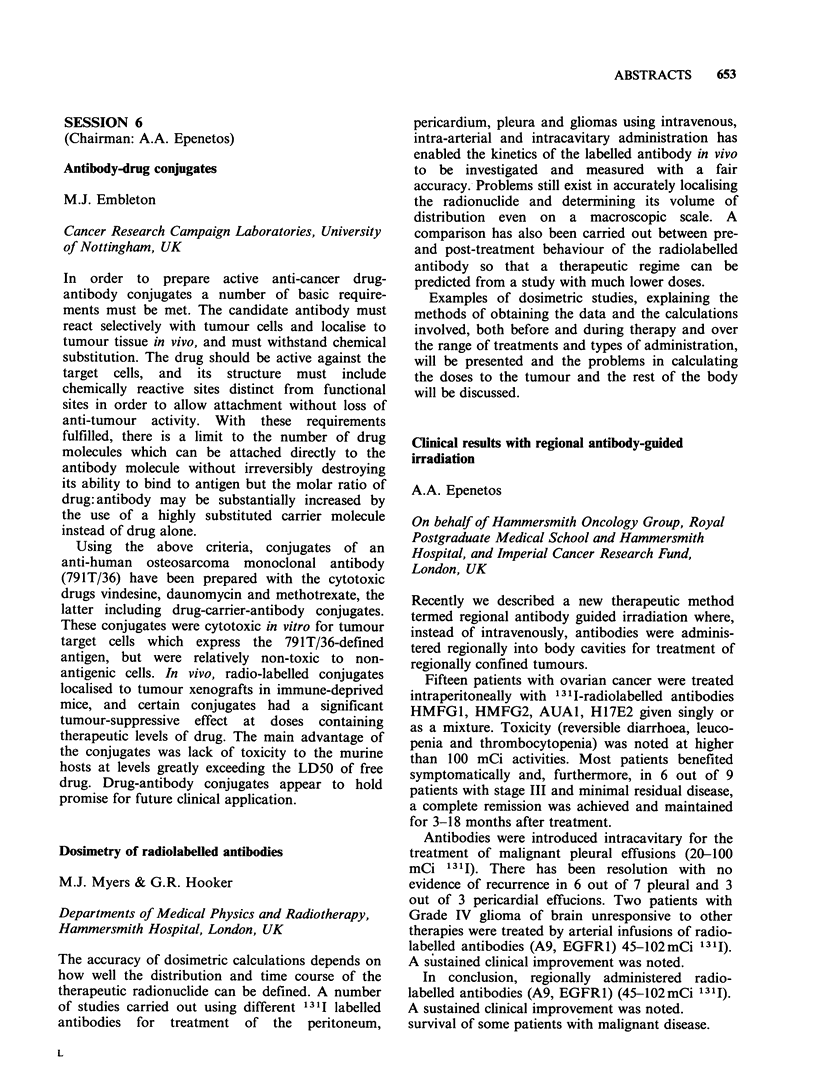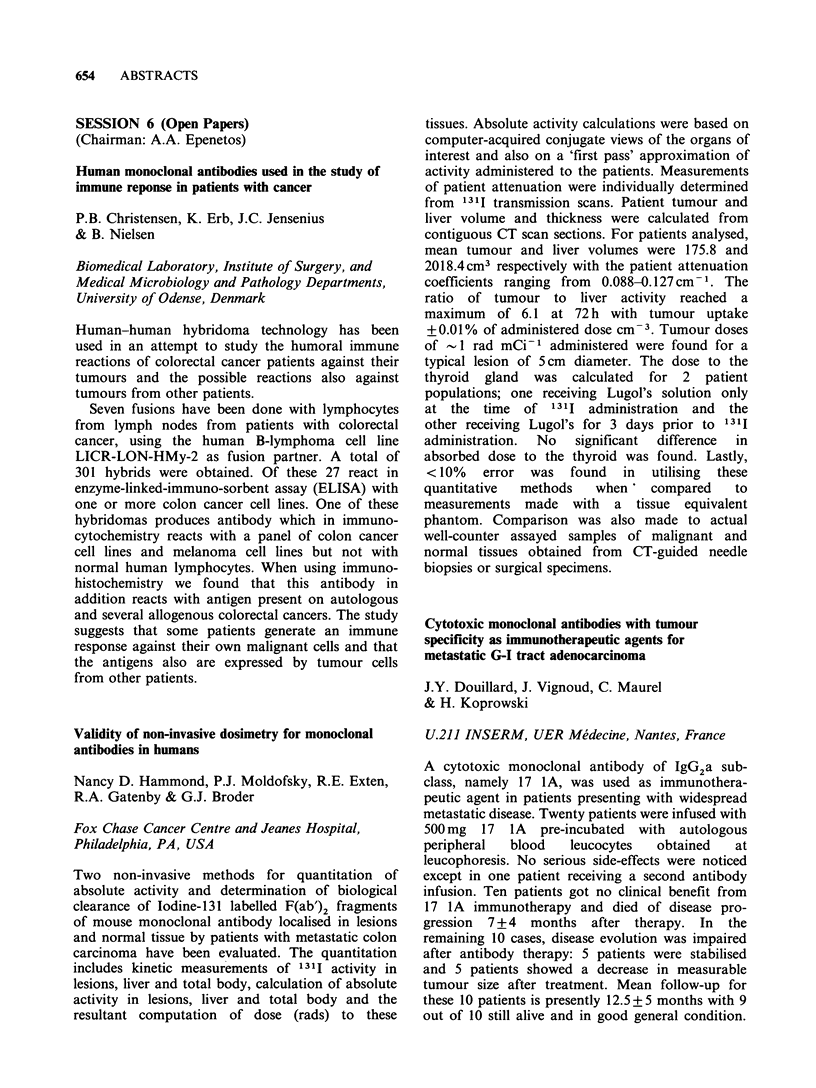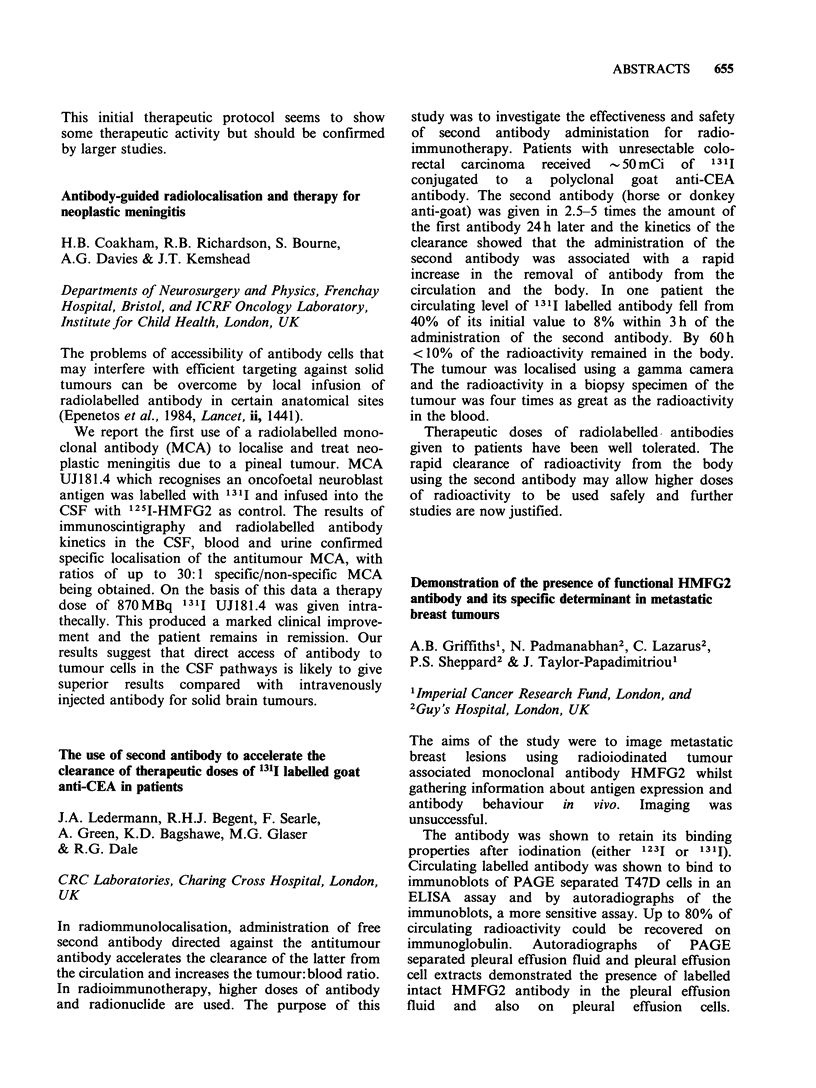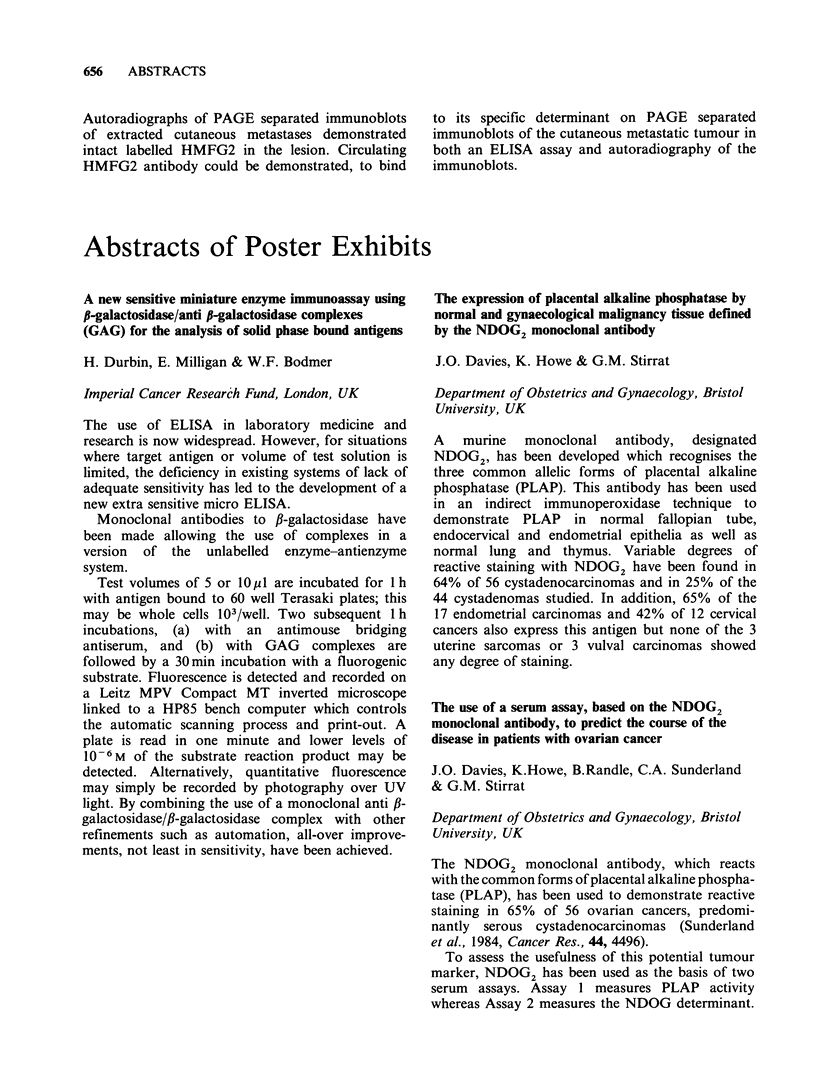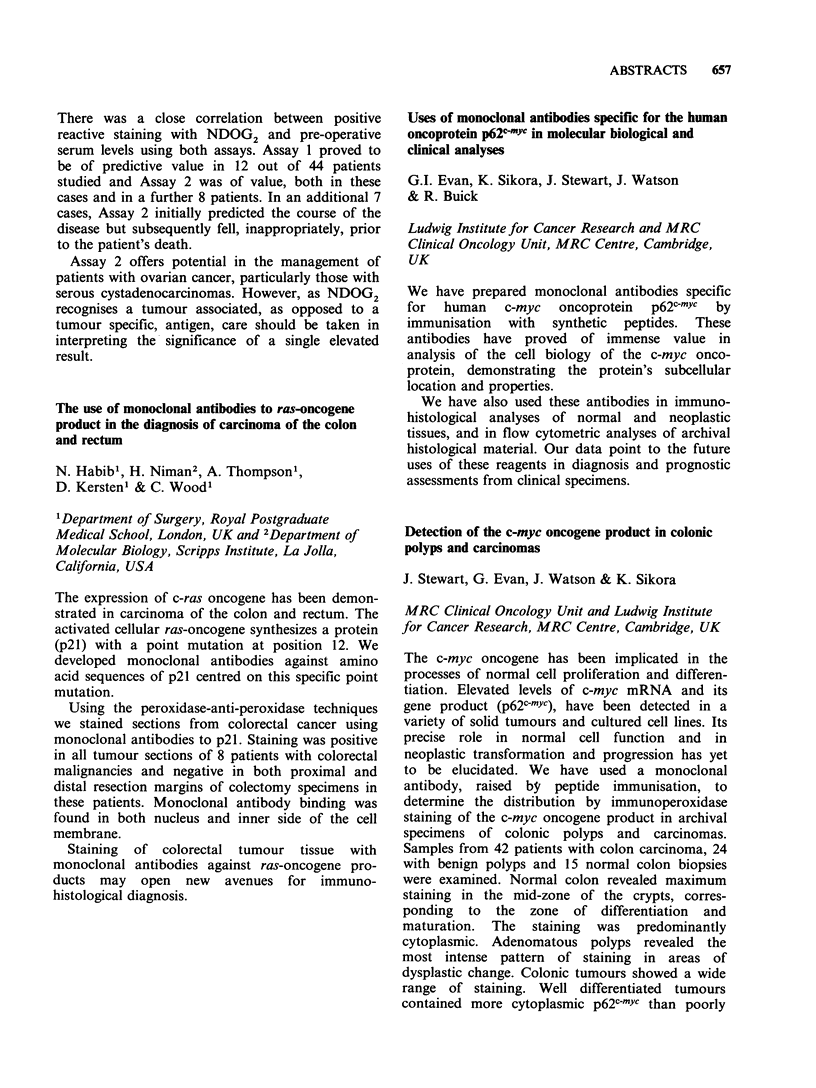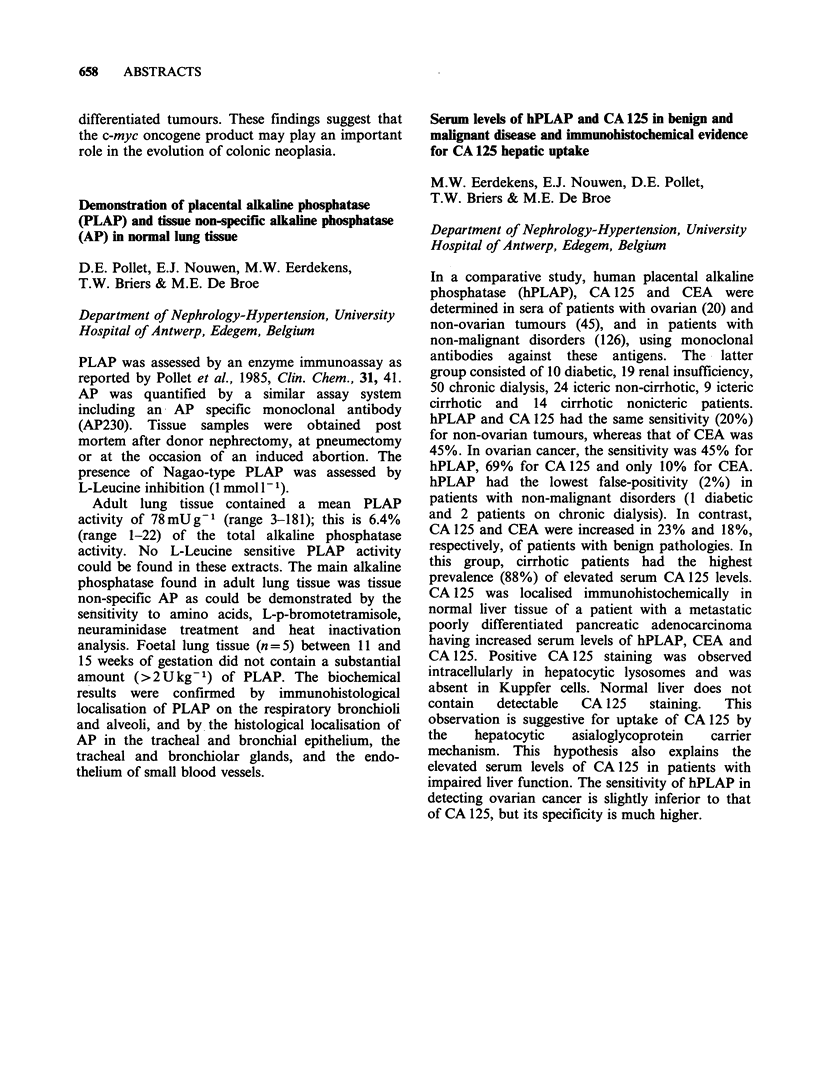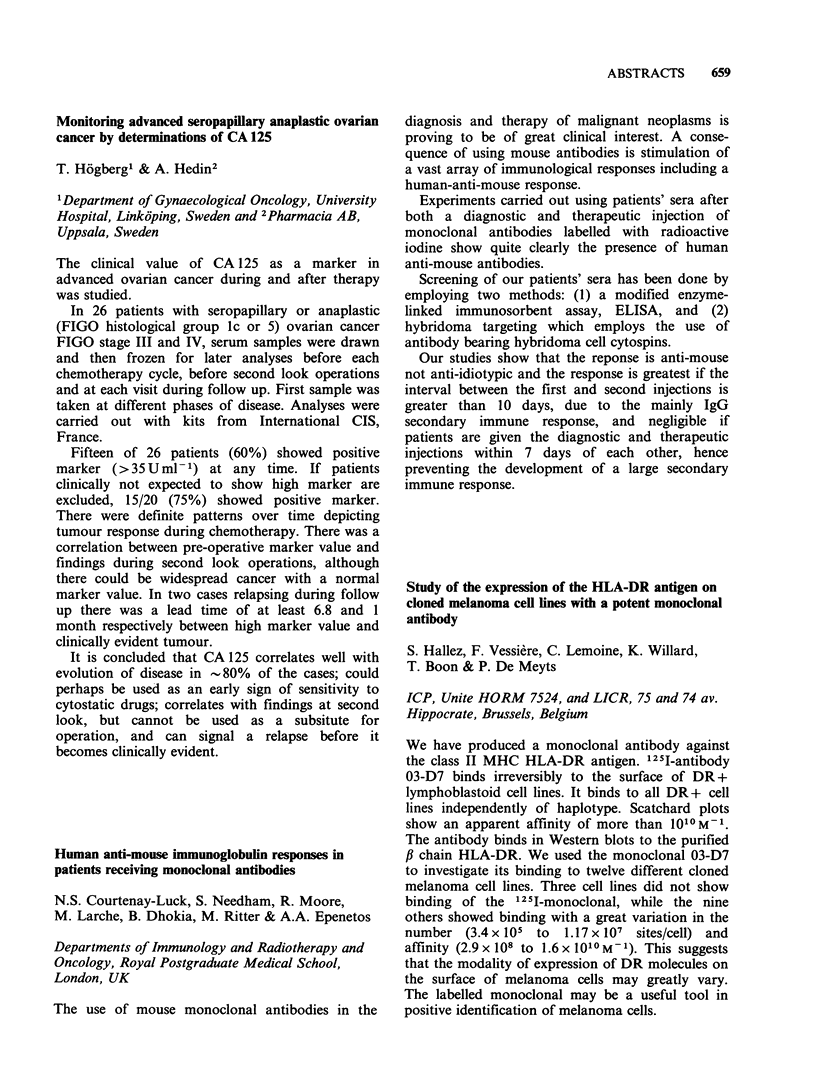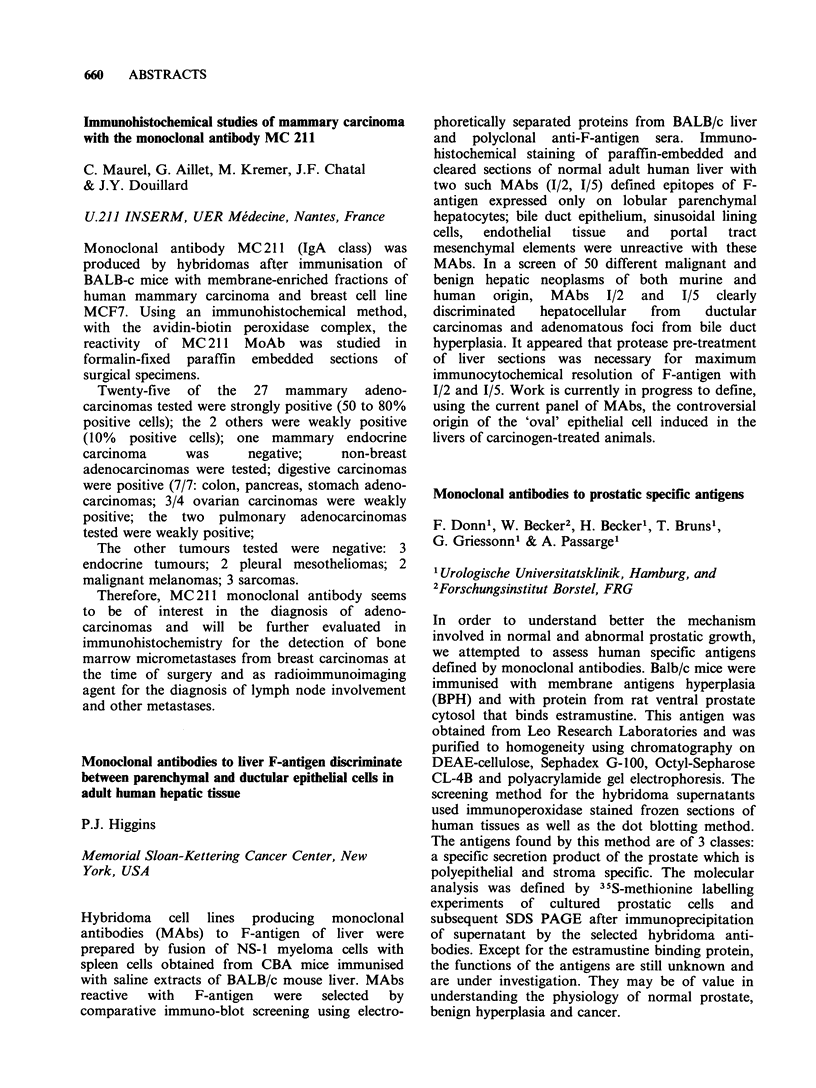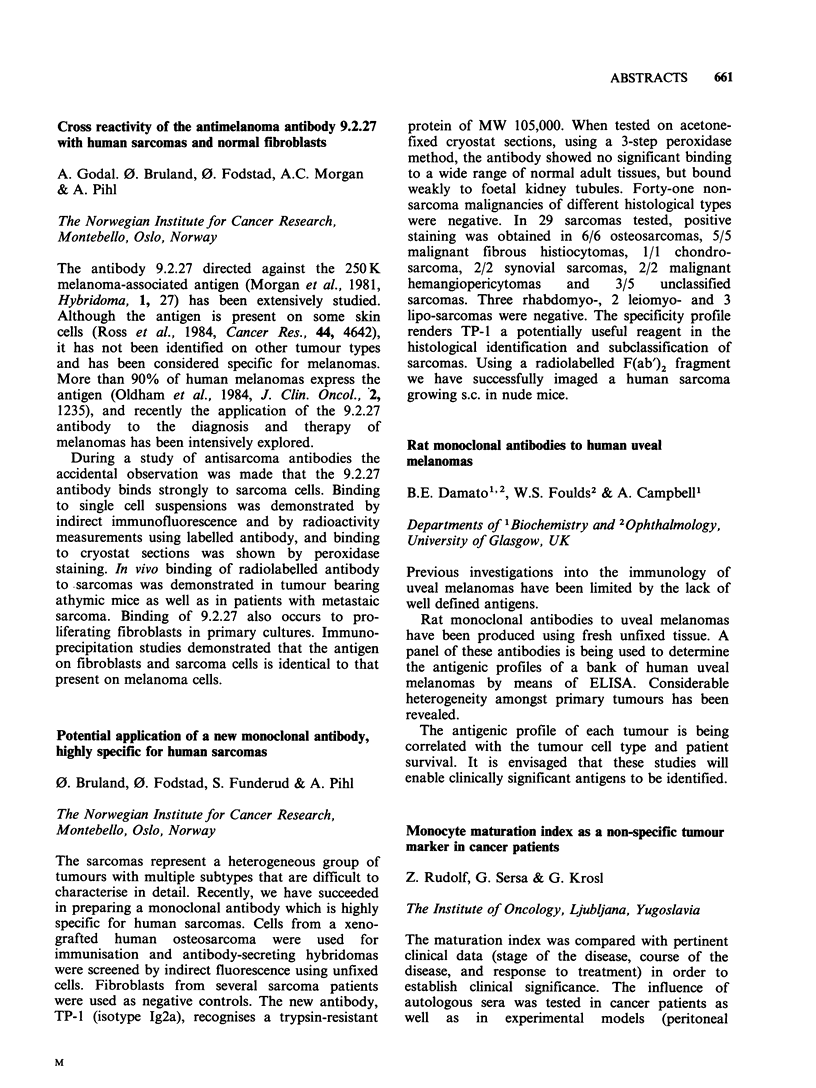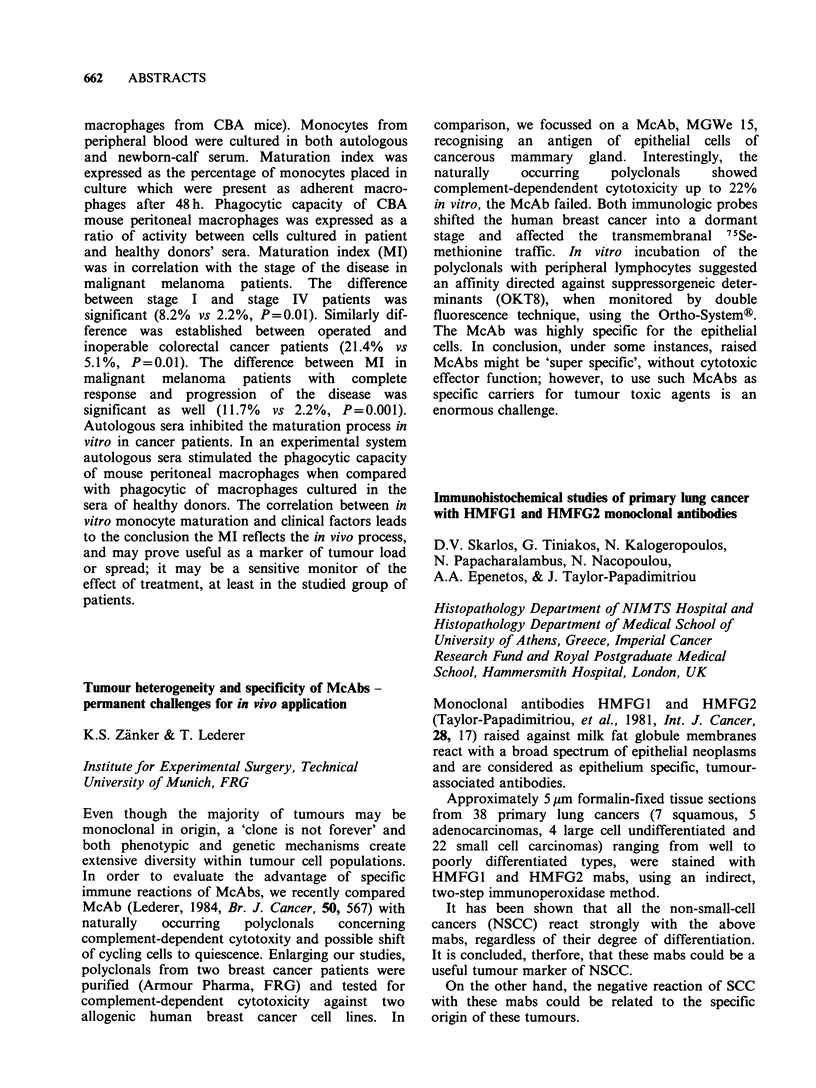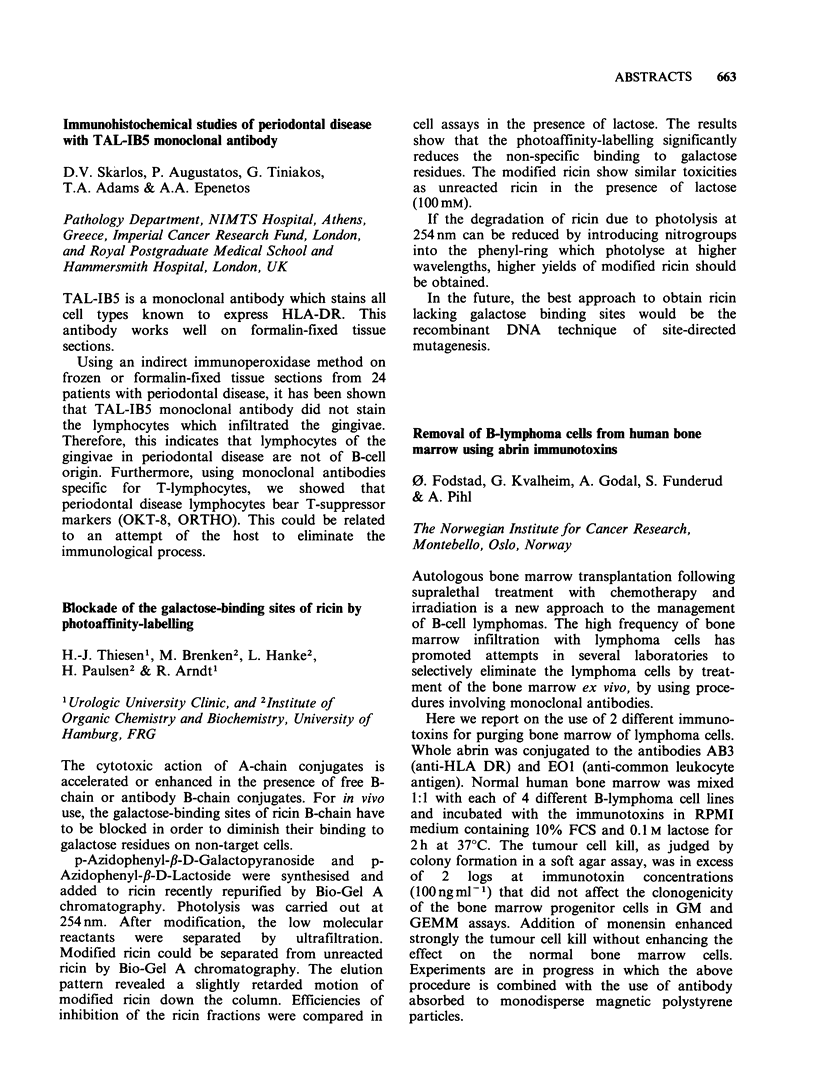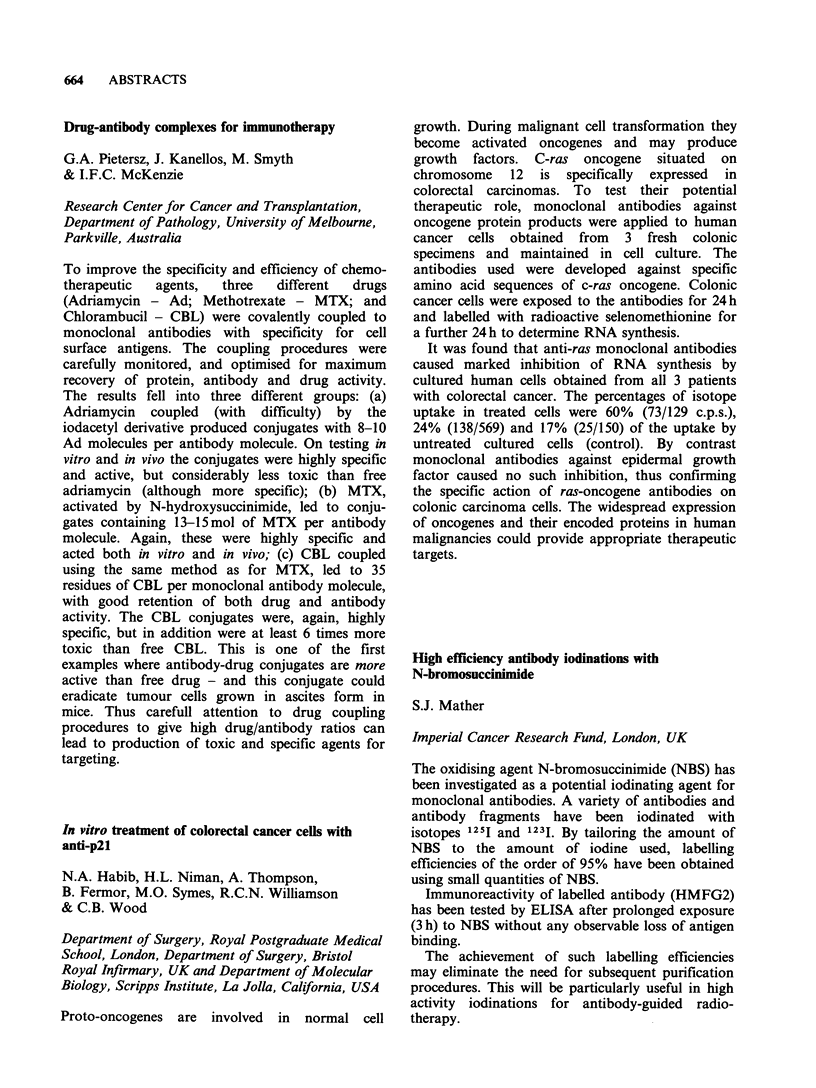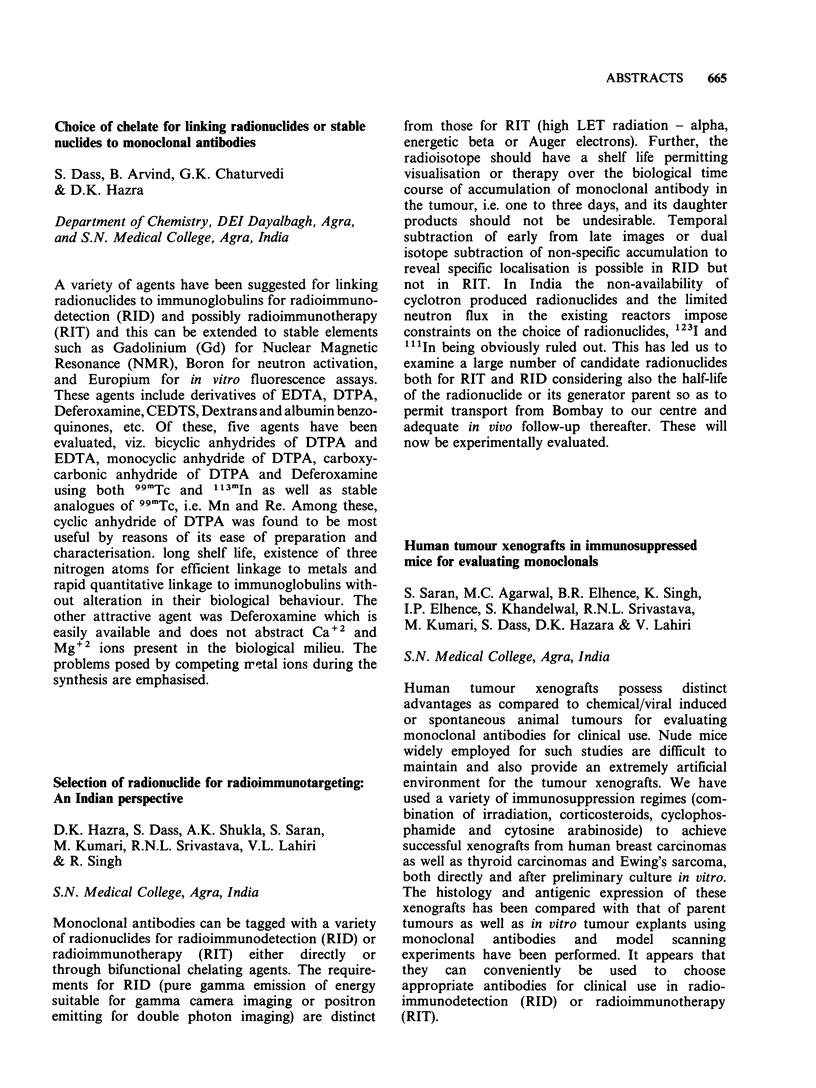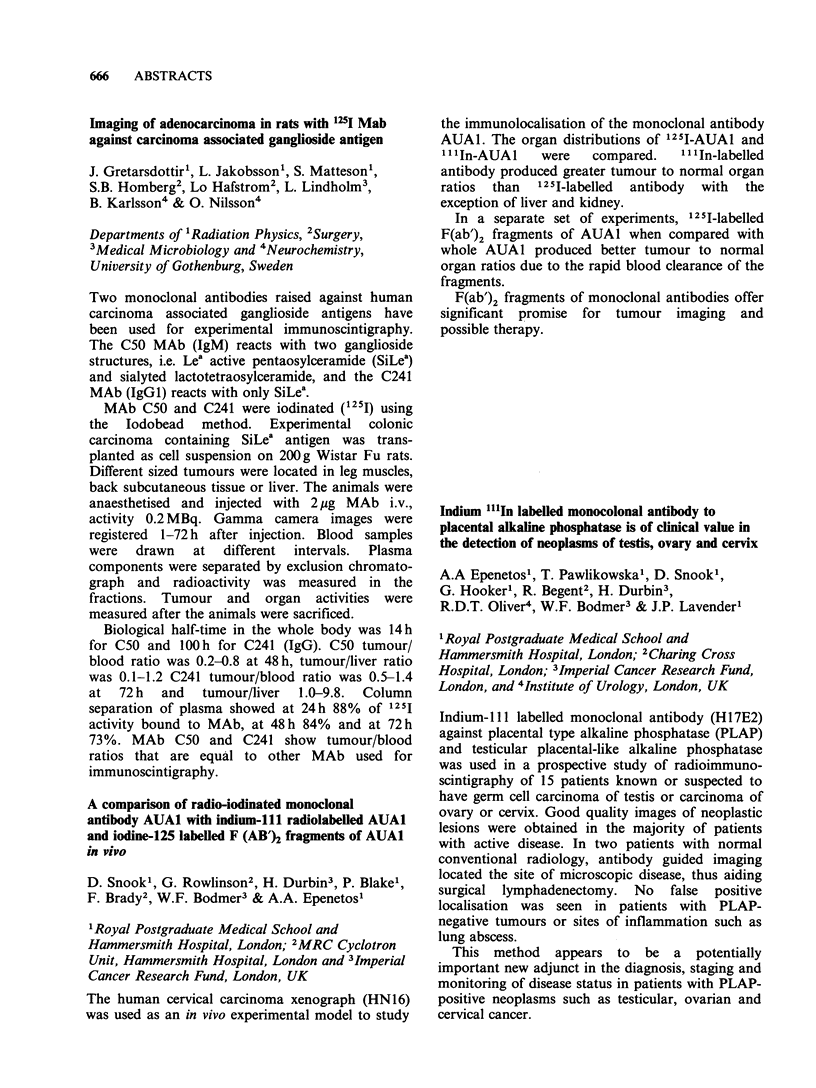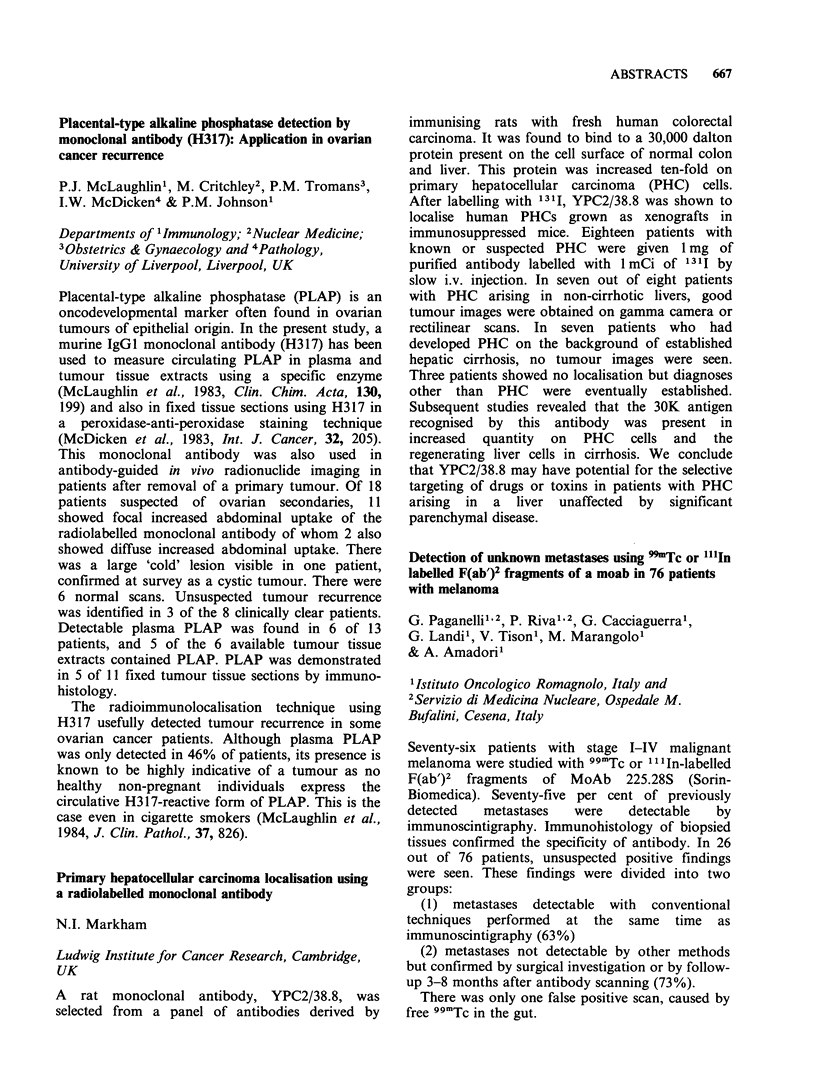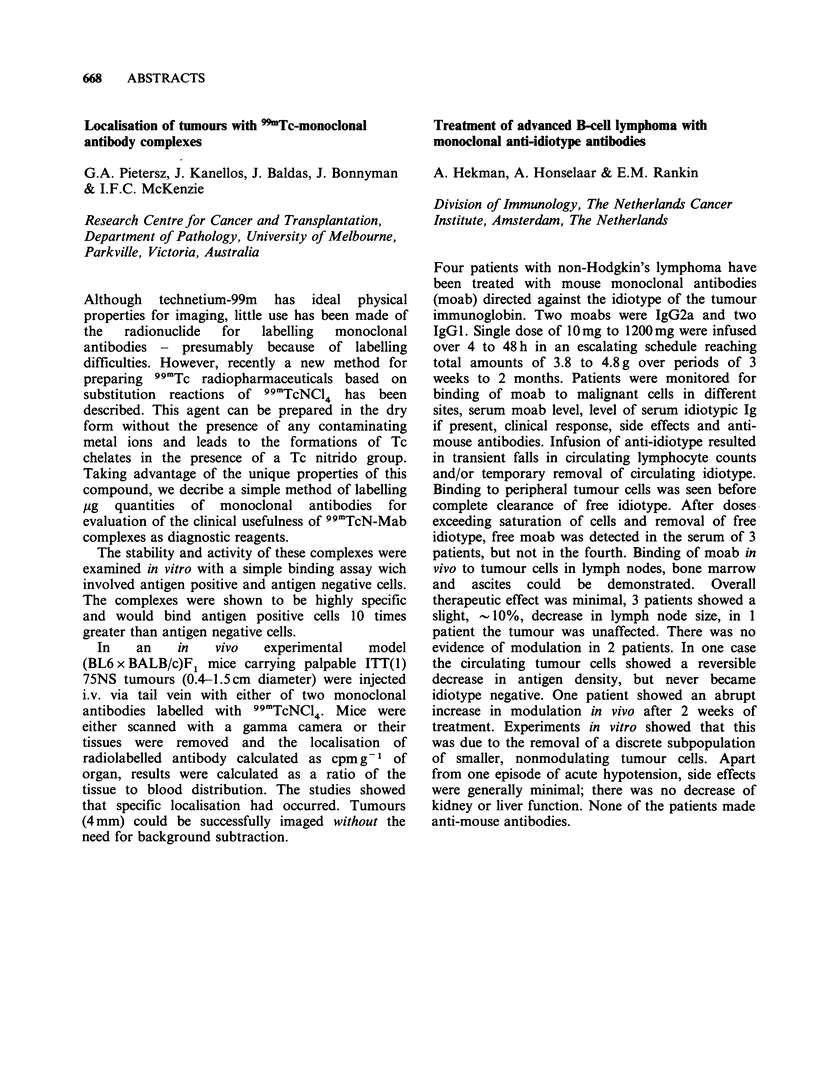# Advances in the applications of monoclonal antibodies in clinical oncology. University of London Royal Postgraduate Medical School. 8th-10th May, 1985. Abstracts.

**DOI:** 10.1038/bjc.1985.240

**Published:** 1985-10

**Authors:** 


Br. J. Cancer (1985), 52, 641–668

### Advances in the applications of monoclonal antibodies in clinical oncology

University of London Royal Postgraduate Medical School

*Held in the Wolfson Institute, 8th–10th May, 1985*

### Abstracts of Oral Presentations

### SESSION 1
(Chairman: K.E. Halnan)

### The monoclonal antibody revolution

J.H. Humphrey

*Royal Postgraduate Medical School, London, UK*

Many attempts (some successful) have been made in the past to obtain tumour-specific antibodies by immunizing animals with tumour cells or extracts followed by removal by absorption of antibodies reactive with the corresponding normal cells. However, the amount of residual specific antibody represented a minute proportion of the remaining Ig and other proteins present in an antiserum. Specificity of labelling was difficult to achieve, and the preparations were liable to be highly immunogenic.

Monoclonal antibodies have changed all this. A large proportion of the Ig secreting cells in a sample from an immunized mouse or rat can be fused with myeloma cells selected to possess very active potential Ig-secretion machinery but by themselves secreting little or no Ig of their own. If a reasonably simple screening method is available, it is possible to select hybridoma secreting antibodies with a desired specificity, even if these occurred only rarely in the starting population. It is becoming possible to predict which determinants on a complex antigen will be most immunogenic, and to design antigens so as to elicit antibodies against attached chosen structural determinants (when these are known).

Course Organisers: A.A. Epenetos & K.E. Halnan, Royal Postgraduate Medical School and Hammersmith Hospital, London W12 0HS, UK.

Course Secretary: A. Freemantle, Department of Medical Physics, Royal Postgraduate School and Hammersmith Hospital, London W12 0HS, UK.

The organisers are grateful to the Imperial Cancer Research Fund for their support.

Although it is not possible to predict in advance what will be the Ig subclass or the affinity for the selected antigen of any particular hybridoma, with persistence and some luck hybridomas secreting Ig of the desired subclass and affinity can be obtained.

The essential feature of monoclonal antibodies is their goodness of fit with the shape of quite small tertiary structures exposed at the surface of the antigen. This allows exquisite specificity, but may result in unexpected cross-reactions.

### SESSION 2
(Chairman: S.E. Order)

### Juggling with immunoglobulin genes

L. Luzzatto

*Department of Haematology, Royal Postgraduate Medical School, London, UK*

Differentiation of individual cell types in the course of ontogenesis is associated with differential expression and repression of genes. In the majority of cases this is brought about by a variety of complex regulatory mechanisms operating in cells that have identical genomes. In this respect, the regulation of immunoglobulin (Ig) genes appears to be exceptional, because it is effected by the construction of functional genes from DNA regions that are separate from each other in the germ-line genome. The assembly of the Ig genes takes place through an orderly sequence of events which are part of the developmental programme of lymphoid cells of the B-lineage. This process makes each individual clone of B cells uniquely different from every other B cell, and this is precisely what has made the development of monoclonal antibodies possible. Very recently it has become clear that a similar process underlies the assembly of the T cell

© The Macmillan Press Ltd., 1985

receptor during the ontogenesis of T cells. The molecular basis for Ig genes assembly can be defined, with due consideration to the phenomena of allelic exclusion and class switch. The analysis of Ig gene rearrangements has turned out to be especially useful for the characterization of leukaemic cell clones. Whereas most characteristics of differentiated cells are appropriately regarded as phenotypic, the Ig produced by B cells and the receptors produced by T cells correspond to genetic changes which have taken place in them at the somatic level. This is a new concept in somatic cell genetics, and it is likely to offer a useful model for an understanding of the role of somatic mutation and recombination in cell differentiation and possibly in oncogenesis.

### Progress in the development of human monoclonal antibodies

K. Sikora

*Ludwig Institute for Cancer Research, Cambridge, UK*

Human monoclonal antibodies against molecules expressed on tumour cell surfaces have proven difficult to construct. Their advantage in revealing new antigenic determinants and their potential for repeated clinical use in therapy have resulted in considerable investment in their production. Major problems include the slow growth rate of human myeloma lines, their low hybridisation frequency and their continued secretion of immunoglobulin coded for by genes of the parental myeloma, resulting in mixed immunoglobulins. There are now over 15 lines available for hybridisation.

We have obtained biopsies from 240 patients with a variety of tumour types and fused lymphocytes from either regional lymph nodes or within the tumour with the lymphoblastoid line LICR.LON.HMy2. Cloned hybrids were obtained from a total of 37 patients. Hybridomas grew rapidly and could be tested for new immunoglobulin secretion. Antibodies were screened by binding activity on the most appropriate tumour cell line and subsequently by immunofluorescence. Twelve antibodies have been found to bind to cell surface antigens on tumour cell lines. Two were evaluated for radioimmunolocalisation. Successful images were obtained in patients with glioma and carcinoma of the bronchus. Recent advances in peptide immunisation, both *in vivo* and *in vitro*, may provide new ways to produce human antibodies of defined specificity.

### Radioactive antibodies in cancer detection and therapy: Experimental and clinical findings

D.M. Goldenberg

*Center of Molecular Medicine and Immunology, University of Medicine and Dentistry of New Jersey, Newark, NJ, USA*

Radioimmunodetection is the method of cancer diagnosis using radiolabelled tumour associated polyclonal or monoclonal antibodies. The standard radionuclide used to date has been I-131. $^{111}$In and Tc-99 have recently been introduced but as yet have undesirable RES accretion. Studies at our centre with conventional goat and murine monoclonal antibodies against carcinoembryonic antigen (CEA), alpha-foetoprotein (AFP), human chorionic gonadotrophin (HCG), prostatic acid phosphatase (PAP), colon-specific antigen-p (CSAp) and Reisfeld's high mol. wt melanoma antigen (9.2.27) have a sensitivity of 90%. In 11 out of 51 patients with colorectal cancer, occult cancer was detected and was later confirmed by other methods, achieving a lead time of up to 40 weeks. Radioimmunodetection revealed all tumours whilst CAT and NMR scanning disclosed 37.5% and 50% respectively. Image resolution ranged between 1.5–2.0 cm tumours. In pancreatic cancers, single photon emission tomography improved imaging.

$^{131}$I anti-CEA IgG was able to retard tumour growth of GW-39 human colonic carcinoma xenografts in animals. Therefore, a clinical Phase I/II trial of radioimmunotherapy has begun. Patients will receive between 25–75 mCi $^{131}$I labelled anti-CEA or anti-AFP antibodies weekly for 4 weeks.

Current results indicate a blood $T\frac{1}{2}$ of 24–36 h and the formation of circulating immune complexes.

### Antibody guided irradiation of cancer

S.E. Order

*Department of Radiation Oncology, The Johns Hopkins Oncology Centre, Baltimore, MD, USA*

Radiolabelled $^{131}$I antiferritin will selectively target tumours that synthesise and secrete ferritin, hepatoma, Hodgkin's disease, etc. In an H42E rat hepatoma, the 'biologic window' that allows selective targeting without normal tissue uptake has directly correlated with neovasculature and ferritin synthesis. In dose escalation studies in hepatoma tumour saturation was determined by volumetric calculations (mean vol. 1000–1500 ml) and radioactive concentration counts (6–12 $\mu$Ci mg$^{-1}$) and has shown that 30 mCi (10 mCi mg$^{-1}$IgG) saturated the tumour. The 4 day tumour effective half life led to a second dose of 20mCi and achieved a 10–12 Gy tumour dose. The toxicity was thrombocytopenia. Volumetric reconstruction of serial CAT scans allowed quantitation of tumour volumes for evaluation of remission. Administration of antiferritin from different species was associated with different tumour effective half lives (rabbit, pig, monkey, bovine 3–4 days) (goat, sheep 2 days), and avoided anti-antibody reactions. In a 105 hepatoma patient experience 48% of the patients had at least partial remission, and 4% of these patients complete remission. One patient remains disease-free in complete remission, 3 years and 3 months, and another patient in partial remission, 5 years and 5 months. Fifteen percent of remitted patients are beyond 2 years.

In Hodgkin's disease, in MOPP-ABVD failures, $^{131}$I antiferritin has a 40% partial remission rate and a 70% remission of B symptoms. Thrombocytopenia occurred earlier due to limited marrow reserve.

### SESSION 3

(Chairman: A.J. Munro)

### Monoclonal antibodies to leucocyte and thymic antigens

M.A. Ritter

*Department of Immunology, Royal Postgraduate Medical School, London, UK*

Monoclonal antibodies to molecules on the cell surface membrane of human leucocytes have provided much information on developmental and functional subsets of haemopoietic cells, as well as on the function of the molecules themselves. For example, for T lymphocytes the identification of the cell surface molecules T1, T3, T4, T6, T8, and T11 has made it possible to study intra-thymic T cell ontogeny, to identify functionally mature subpopulations of recirculating peripheral T lymphocytes and to study some of the molecular events involved in T cell activation. Clinically, monoclonal antibodies to leucocytes have been useful both in assessing immune status and in analysing haemopoietic malignancies. Normal haemopoietic differentiation depends upon an interaction between the progenitor cells and their microenvironment. We have recently raised monoclonal antibodies to molecules in/on the epithelial component of the human thymus microenvironment, and using these can identify several cell surface and secretory products that may be responsible for progenitor/lymphocyte positioning, migration and differentiation within the thymus. We are currently using these reagents to analyse the thymic microenvironment in myasthenia gravis. Preliminary data indicate that whereas in patients with thymic hyperplasia it is the subcapsular/medullary epithelium that is proliferating, in thymoma cases it is the cortical epithelium that is involved in the neoplasia. (Work in collaboration with: Drs J. Newsom-Davies, N. Wilcox, J. Janossy, Royal Free Hospital, London; Dr H.-J. Schuurman, University Hospital, Utrecht).

### Breast epithelial differentiation antigens

J. Burchell, J. Bartek & J. Taylor-Papadimitriou

*Imperial Cancer Research Fund, London, UK*

In the haemopoietic system, the study of normal differentiation has greatly aided in the management of many leukaemias. In an attempt to apply a similar approach to the study of breast carcinomas, the differentiation of mammary epithelial cells is being investigated. To facilitate this study, monoclonal antibodies have been produced to (1) human B casein, a milk protein which is a unique product of terminally differentiated breast epithelium, (2) cytokeratins, which are expressed exclusively by epithelial cells, the profile of expression being characteristic of a specific epithelial cell type, and (3) a large mol. wt mucin-like component present in human milk fat globule which is recognised by monoclonal antibodies HMFG-1 and 2. The HMFG-1 antigen is highly represented on this mucin and on lactating breast, and its presence has been associated with the decrease growth potential of normal breast epithelium *in vitro*. In contrast, the HMFG-2 antigen is poorly represented on the mucin, but is strongly expressed by most breast cancer cell lines and ductal carcinomas and its presence in the serum of advanced breast cancer patients may be associated with a bad prognosis.

These antibodies are now being used to characterise breast epithelial cells *in vitro* cultured on plastic and in collagen gels. Additionally, the HMFG-1 and 2 antibodies have been found to have practical uses in the clinic including (1) the identification of carcinoma cells in serous effusions, (2) in *in vivo* imaging of carcinomas, especially ovarian, and (3) targeting of therapeutic doses of radiation.

### SESSION 3 (Open Papers)
(Chairman: A.J. Munro)

### Enzyme amplification and oncological markers

C.H. Self

*Department of Chemical Pathology, Royal Postgraduate Medical School and Hammersmith Hospital, London, UK*

Enzyme amplification provides a means of developing highly sensitive and rapid assays for oncological markers. The assays may be made colorimetric and thus simple to read.

Amplification is achieved by causing the enzyme to be determined to produce a catalytic activator for a secondary enzyme system, the activity of which is measured. Thus the catalytic effect of the enzyme to be determined is multiplied by the catalytic effect of the activator it produces. The measured activity is correspondingly large.

The enzyme activity which is amplified may be either that of the analyte itself, when this has a suitable enzymatic activity, or that of an enzyme conjugate in, for example, ELISA systems. The former case has been demonstrated by the application of enzyme amplification to a monoclonal antibody-based capture assay for placental alkaline phosphatase. This has resulted in a much more sensitive and rapid assay compared to conventional methods. The application of enzyme amplification to ELISA systems has meant that a very wide range of materials of oncological importance may now be assayed by amplified systems. The advantages of this approach have been shown, for example, by the determination of prostatic acid phosphatase by enzyme amplified ELISA.

### Application of monoclonal antibodies to the characterisation of acute leukaemias.

E. Matutes, A. Parreira, C. Andrews, V. Moss, A. Karabyn & D. Catovsky

*MRC Leukaemia Unit, Royal Postgraduate Medical School, London, UK*

Immunological analysis of haemopoietic precursors by using cell lineage specific monoclonal antibodies (McAb) has led to a better recognition of leukaemic cells and has increased the diagnostic possibilities of otherwise unclassifiable leukaemias. In this study we describe the phenotypic characterisation of blast cells from a series of over 200 patients with acute leukaemia and blast transformation of chronic granulocytic leukaemia (CGL) and myelofibrosis (MF) by an indirect immunofluorescence technique using the following McAb: (1) LICR-LON 10-antiglycophorin A- and GERO – against the RBC group Gerbich – to recognise erythroid cells; (2) AN51-antiplatelet glycoprotein (gp) Ib-; C17-antiplatelet gp IIIa and J15-antiplatelet gp IIb/IIIa – to identify megakaryocytic precursors; (3) My9 — against myeloid and monocytic cells – and 3C5 – reactive with early myeloid and lymphoid percursor cells; (4) terminal deoxynucleotidyl transferase (TdT); J5 (anticommon ALL); anti-B4 (early B-cells); 3A1 (anti-p40) and OKT17, to identify early B and T cell lymphoblasts. Data will be presented on the value of this approach in: (a) the characterisation of some 'undifferentiated' leukaemias as proliferations of erythroid and megakaryocytic precursors; (b) the identification of leukàemias with mixed cellular components (e.g. myeloid and megakaryocytic, etc.); (c) the distinct reactivity of the McAb My9 and 3C5 in the various types of acute myeloid leukaemia according to the FAB classification, e.g. My9+, 3C5+ in myelobasts (M1) and My9+, 3C5− in monoblasts; (d) the classification of the T cell malignancies according to the reactivity with TdT, 3A1 and OKT17 in early thymic (TdT+, 3A1+, OKT17−); thymic (TdT+, 3A1+, OKT17+) and post-thymic (TdT−, 3A1−, OKT17+) proliferations.

### Immunohistochemical localisation of hPLAP, CA 125 and CEA in normal and neoplastic human tissues and the foetus

E.J. Nouwen, M.W. Eerdekens, T.W. Briers, D.E. Pollet & M.E. De Broe

*Department of Nephrology-Hypertension, University Hospital of Antwerp, Edegem, Belgium*

Human placental alkaline phosphatase (hPLAP), CA 125 and CEA were localised on adjacent paraffin sections using monoclonal antibodies (E6, OC 125 and the Hybritech antibody, respectively) and an indirect avidin-biotin-peroxidase staining procedure. On 8 malignant lung tumours, 4 were positive for hPLAP, 6 for CA 125 and 7 for CEA. All 13 normal lungs were positive for the three antigens. hPLAP staining was present in the respiratory bronchioli and alveoli. CA 125 was observed in the trachea, bronchi, respiratory glands, terminal bronchioli and pleural mesothelium. CEA staining was present in the trachea, bronchi, respiratory bronchioli and alveoli. In the foetal lung, hPLAP could not be demonstrated. CA 125 was present in the foetal trachea, bronchi, respiratory glands and mesothelium. On 23 malignant ovarian tumours, 18 were positive for hPLAP, 22 for CA 125 and 9 for CEA. CA 125 staining was in general more abundant than hPLAP staining. Two of 13 normal ovaries were positive for hPLAP and 12 for CA 125, whereas CEA staining was absent. CA 125 staining in the normal ovary was present in the surface epithelium, local proliferations of this epithelium, invaginations and germinal inclusion cysts. hPLAP staining was observed only in some of the germinal inclusion cysts. The foetal ovary was positive for hPLAP, but negative for CA 125 and CEA. Normal tuba was positive for hPLAP and CA 125, but negative for CEA. On 18 malignant gastrointestinal tumours, 10 were positive for hPLAP, 6 for CA 125 and 18 for CEA. hPLAP and CA 125 staining was absent in 12 normal gastrointestional tissues, but CEA staining was always present. The foetal oesophagus was positive for CA 125. On 7 malignant breast tumours, 1 was positive for hPLAP, 6 for CA 125 and 4 for CEA.

### Immunoreactivity of monoclonal anti-melanoma antibodies iodinated by different methods

S. Matzku & H.J. Sinn

*Institute of Nuclear Medicine, German Cancer Research Centre, Heidelberg, FRG*

Six monoclonal antibodies (MAbs) were iodinated using chloramine T, IODO-GEN or N-bromosuccinimide to achieve increasing levels of iodine substitution. Immunoreactivity was characterised by sequential absorption, by Lineweaver-Burk-type extrapolation to binding at infinite antigen (melanoma cell) concentration, and by Scatchard plots. Our findings can be summarised as follows:

(1) Immunoreactivity depended on iodine substitution ratios rather than on the iodination method, provided that mild reaction conditions were adopted.
(2) MAbs displayed considerable individuality with respect to the level of substitution they tolerated.
(3) Iodination was found to be an all or none effect reducing the proportion of antibody molecules binding at antigen excess, or a gradual effect reducing binding affinity. Reduced immunoreactivity was clearly reflected by a poor performance of labelled MAbs *in vivo*, e.g. in immunoscintigraphy.

### Iodination of monoclonal antibodies for diagnosis and radiotherapy using a convenient one vial method

H. Haisma, J. Hilgers, W. Den Otter & V.R. Zurawski, Jr.

*Centocor, Inc., Malvern, USA, Netherlands Cancer Institute, Amsterdam, and Institute of Pathology, Utrecht, The Netherlands*

The use of monoclonal antibodies for diagnosis and therapy of cancer is well established. $^{123}I$ and $^{131}I$ are the isotopes of choice in most cases. The use of radiolabelled antibodies is restricted to specialised centres for the iodination of antibodies is rather complicated. We have developed a convenient system using a one vial method for iodination of antibodies for diagnosis and therapy. A vial previously coated with iodogen is used as a reaction vessel. Iodination and separation of bound and free iodine using AGI-X8 (Dowex) ion exchange resin are both accomplished in this vial. Using four different monoclonal antibodies reactions incorporated $90 \pm 4\%$ of the iodine which was added. Approximately 90% of labelled antibody was recovered in each case. The monoclonal antibody OC125 was labelled to specific activities up to 25 mCi $mg^{-1}$ with immunoreactivities of $82 \pm 2\%$ using $^{125}I$ and $66 \pm 5\%$ using $^{131}I$. As the radioiodination is done in one sealed vial and takes $<10$ min, this procedure is safe and can be performed in any nuclear medicine laboratory. The final product, which is sterile and pyrogen-free, is suitable for diagnostic and radiotherapeutic applications. We anticipate that this method has great implications for the use of iodinated antibodies for imaging and therapy making radioimmunodetection and antibody guided radiation therapy less restricted to specialised centres.

### NMR relaxation time in nude mice bearing human colorectal adencarcinoma injected with MoAb 19.9 coupled with gadolinum-DTPA as contrast agent

C. Curtet[1], J. Grzyb[2], C. Tellier[1], J.Y. Douillard[1] & J.F. Chatal[1]

[1]*INSERM U.211, UER Médecine &* [2]*CEA de SACLAY, Nantes, France*

Gadolinium-DTPA MoAb 19.9 are presently being investigated for their potential use as tumour-specific (NMR) imaging agents.

The strongly paramagnetic gadolinium complex reduces hydrogen proton relaxation. The metal ion ($Gd^{3+}$) needs to be chelated with a suitable ligand (DTPA) to decrease the toxicity, and to enable coupling with MoAb 19.9 which is direct against specific tissues (human colon adenocarcinoma SW 1116, HT 29, and HRT 18).

The effect of the paramagnetic compound on proton relaxation $T_1$ and $T_2$ was measured in aqueous solutions for different concentrations at 60 and 90 MHz using the Bruker NMR spectrometer.

Pharmacokinetics were performed with 153 Gd labelled compound (0.3 $\mu Ci \mu g^{-1}$) to study the biodistribution at days 1, 2 and 5. The best binding in the tumour was observed at days 1 and 2.

Finally, we injected Gd-DTPA MoAb 19.9 ((Gd) 1 mM, (DTPA) 1 mM, (MoAb) 0.06 mM) in mice bearing HRT 18 tumour.

A 20% difference was noted relative to controls.

### A prognostic index for primary breast cancer incorporating staining with monoclonal NCRC 11

I.O. Ellis, M.R. Williams, J.H. Todd, C.W. Elston & R.W. Blamey

*Department of Histopathology, Queens Medical Centre, and City Hospital, Nottingham*

A monoclonal antibody NCRC 11 has been raised against breast carcinoma. It has a similar immunohistological staining distribution to epithelial membrane antigen (EMA).

The prognostic importance of monoclonal staining with NCRC 11 antibody has been demonstrated on a series of 126 primary tumours.

A prognostic index for primary operable breast cancer has been previously described. This is dependent on size, grade and lymph node stage of the primary tumour.

In this study we confirm this prognostic power on 379 tumours and further investigate the relationship with grade, ER, lymph node status and size of primary tumour.

NCRC 11 monoclonal staining is found to relate to both grade and ER (intrinsic factors) but not to stage or size (time dependent factors). It has independent prognostic power and has been incorporated into a new prognostic index.

### SESSION 4

(Chairman: J.P. Lavender)

### Radiochemistry of DTPA-coupled monoclonal antibodies

D.J. Hnatowich

*University of Massachusetts Medical Center, Worcester, MA, USA*

The majority of investigators studying radiolabelled monoclonal antibodies for diagnosis and therapy of cancer label with radioisotopes of iodine. An attractive alternative approach is to first covalently attach a strong chelating group such as diethylenetriaminepentaacetic acid (DTPA) so that the antibody may be labelled later with one of several metallic radionuclides. Using antibodies coupled with DTPA via the cyclic anhydride, we have evaluated the chemistry of labelling with three radionuclides: $^{111}In$ and $^{99m}Tc$ for tumour diagnosis and the beta-ray emitting $^{90}Y$ for tumour therapy. The important property of label stability in 37°C serum was determined *in vivo* and *in vitro* by methods involving high performance liquid chromatography, affinity and open-column gel filtration chromatography.

Our results show that the attached DTPA groups do not dissociate from the antibody in serum environments. Furthermore, when DTPA-coupled antibodies are labelled with either $^{111}In$ or $^{90}Y$, the label is unstable only to transcomplexation to transferrin. The rate of this dissociation is about 8–9% of the activity in serum per day. When the DTPA-groups on antibodies are labelled with $^{99m}Tc$, a similar transcomplexation of label to serum proteins is observed: however, in this case, the label is also unstable towards oxidation to pertechnetate.

### Biodistribution and pharmacokinetics of In-111 labelled monoclonal antibody F(ab′)$_2$ fragments in patients

P.W. Doherty

*University of Massachusetts Medical Center, Worcester, MA, USA*

We studied 14 patients who had known sites of tumour, with the antibody 19–9 labelled with $^{111}In$, using the cyclic anhydride approach. Following injection of 1 mg of the fragments labelled with 1–2 mCi of $^{111}In$, biological clearance was evaluated by whole body scanning and organ uptake was quantitated using attenuation correction. Serial blood and urine samples were examined by HPLC and affinity chromatography to determine the chemical form of activity. The clearance of radioactivity from serum was biphasic with a $T\frac{1}{2}$ of 2 and 19 h. Whole body clearance was 160 h with urine loss predominating at 0.26% of injected dose excreted per hour. Regionally, 20% of the activity was present in the liver and 10% in the kidneys with maximum levels occurring at 24 h and did not decrease much over time. Translocation of activity to transferrin occurred at a rate of 9% $day^{-1}$ and antigen antibody complex formation was not a major problem. Good quality images of tumour uptake were obtained in 8 of 12 sites, most obvious at 48 h. Those sites in which uptake was not seen were liver metastases. SPECT imaging was helpful in providing better localization information. Overall, the labelled antibody is quite stable *in vivo* and the distribution of the radioactivity is equivalent to the distribution of the antibody. The major site of catabolism appears to be the liver from which the $^{111}$In is not released to any great extent. These observations have implications for those considering this approach to therapy.

### $^{123}$I labelled antibodies – state of the art

K.E. Britton and M. Granowska

*Department of Nuclear Medicine, St. Bartholomew's Hospital, London, UK*

The monoclonal antibody is a homogeneous gamma globulin with many tyrosine radicals suitable for iodination using the gentle techniques refined for radioimmunoassay over many years. $^{131}$I is unsuitable as the iodine label because its beta emission and long half-life cause high patient radiation when given in a dose sufficient to give good imaging sensitivity and its use should be limited to therapy. $^{123}$I with no beta emission, 13 h halflife and 16 MeV gamma ray energy is ideal for imaging with the gamma camera. It is becoming more widely available and per MCi is half the price of $^{111}$In, the alternative label. It has been shown that $^{123}$I labelled monoclonal antibodies preserve their immunoreactivity whereas chelation for $^{111}$In labelling may cause cross-linking of antibodies, leading to high liver uptake and reduced immunoreactivity. The kinetics of antibody uptake favour a high sensitivity, short-lived radionuclide which gives a good signal. Our original technique of subtracting an early image from a later one has been refined through kinetic analysis of serial images with probability mapping. On the assumption that tissue background is decreasing with time and tumour uptake is increasing with time, serial images may be compared pixel by pixel and the frequency distribution of activity plotted. Using a least squares technique the clusters of pixels showing significant positive or negative deviation with time may be identified and the areas of significant change, at, for example, the $P<0.001$ plotted on a map as a contour over the original data. This objective approach is needed to define that an abdomen is really clear or not of recurrence.

### Clinical decisions based on antibody guided scanning

R.H.J. Begent, F. Searle, A. Green & K.D. Bagshawe

*CRC Laboratories, Charing Cross Hospital, London, UK*

Radioimmunoscintigraphy is capable of locating a variety of malignant tumours. Its place in clinical practice depends on its performance in defined clinical situations where the result determines a decision about patient management.

Since 1980 we have studied patients with a raised serum tumour marker concentration in whom the site of disease was not known but in whom resection of localised tumour might be beneficial. Patients with drug resistant choriocarcinoma and raised serum human chorionic gonadotrophin (hCG) were studied with antibody to hCG. Similar patients with germ cell tumours and raised serum hCG or alphafoetoprotein (AFP) received antibody to hCG or AFP as appropriate. Patients with raised serum carcinoembryonic antigen (CEA) values after apparently complete resection of primary colorectal cancer received antibody to CEA.

Radioimmunoscintigraphy was able to discriminate between localised and disseminated disease and improved the selection of patients for surgery. In some instances the method detected tumour when conventional imaging failed. In these diseases which are usually disseminated at the stage studied, it was possible to select $\sim 10\%$ with localised disease who have obtained sustained complete response as a result of surgical resection.

### SESSION 4 (Open Papers)

(Chairman: J.P. Lavender)

### Monoclonal antibody imaging as an adjunct to radiographic CT in abdominal metastases

P.J. Moldofsky, N.D. Hammond, R.E. Exten, R.A. Gatenby & G.J. Broder

*Fox Chase Cancer Center and Jeanes Hospital, Philadelphia, PA, USA*

In imaging over 50 patients with $^{131}$I labelled $F(ab')_2$ fragments of monoclonal antibodies directed against colorectal carcinoma cell surface antigens we have identified 5 areas of potential utility for this diagnostic method to provide information complementary to that provided by radiographic CT. Approximately 100–500 $\mu g$ of $F(ab')_2$ fragments are labelled by the iodogen method with approximately 37 MBq (1 mCi) $^{131}$I. I.v. administration to patients with known or suspected colorectal carcinoma is followed by daily imaging for up to 7 days. Patients also undergo radiographic CT scanning and either a CT-guided biopsy or surgical biopsy for pathology and immunoperoxidase staining for the colorectal antigen.

Patients have been identified in 5 categories of diagnostic utility where the antibody images provided information beyond that available from CT: (1) Small disease: lymph nodes harbouring microscopic metastases, normal in size (1 cm) on CT, have been detected by antibody imaging. (2) Differentiation of post-operative change from recurrent tumour: despite little change in soft tissue mass at the primary operative site on CT scans 3 months apart, antibody scan indicated recurrent tumour, confirmed at surgery. (3) Localization of recurrence in patients with no identifiable disease by CT but rising serum CEA. (4) Differentiation of types of tumour metastasis in patients with more than one, or unknown, primary. (5) Quantitative evaluation of localization of antibody in tumour, for therapeutic applications. While most sites of disease were localized by CT scans, we have identified 5 categories of additional information provided by the antibody scans that have influenced clinical decisions in these patients.

### Imaging of cutaneous T-cell lymphoma (CTCL) with $^{111}$In and $^{131}$I labelled T101

J.A. Carrasquillo, J.M. Mulshine, J.C. Reynolds, A.M. Keenan, P.A. Bunn, K.A. Foon, R.W. Schroff, A.F. Gazdar, P. Perentesis, M. Horowitz & S.M. Larson

*CC, NCI, BRMP, National Institutes of Health, Bethesda, MD, USA*

T101 is a murine monoclonal antibody (MOAB), IgG2a directed against a pan T-cell antigen present in high concentration in CTCL. $^{111}$In labelling (5 mCi, 1 mg) was performed by a modification of the Krejcarek method (Hybritech, Inc.): $^{131}$I labelling (2 mCi, 1 mg) was performed via the chloramine T method. Six patients received 1 mg $^{111}$In T101 and 3 patients received 1 mg $^{131}$I T101 i.v. by a 2-h infusion. By 24 h all patients receiving $^{111}$In T101 showed uptake in involved nodes as well as previously unsuspected nodes. Nodes as small as 5 x 5 mm were visualised. Skin plaques (6 patients) were negative whereas erythroderma (1 patient) was positive on scan. In addition, prominent liver, spleen and bone marrow uptake was visualised and persisted on serial scans. $^{131}$I T101 showed minimal nodal concentration in 1 patient and none in the other 2 patients. Although early images showed prominent liver, spleen and to a lesser extent bone marrow uptake, serial images showed rapid clearance of $^{131}$I from these organs. One patient received both $^{131}$I and $^{111}$In-labelled T101 with no localisation in involved nodes with $^{131}$I label but prominent areas of nodal uptake and skin (erythroderma) with $^{111}$In T101 (4 days later). Plasma clearance was rapid for all preparations with <20% in the plasma volume by 2 h post-infusion. Whole body retention was prolonged for $^{111}$In *versus* $^{131}$I T101 ($T\frac{1}{2}$ of >9d *vs* <2d). Rapid dehalogenation was observed, with free $^{131}$I excreted in the urine. No toxicity was observed. Modulation of antigen from circulating cells, skin and nodes was seen. The study shows the feasibility of imaging CTCL with $^{111}$In T101 and shows major differences in biodistribution when compared to $^{131}$I T101.

### Radioimmunoscintigraphy (PLANAR + SPECT) as a method in the follow-up of ovarian cancer patients

N. Pateisky[1], K. Philipp[1], H. Bergmann[2], G. Hamilton[3] & J. Burchell[4]

*[1]1st Department of Gynaecology and Obstetrics, [2]Department of Nuclear Medicine, [3]1st Department of Surgery, University of Vienna, Austria; and [4]Imperial Cancer Research Fund, London, UK*

More efficient methods are needed for screening after recurrent disease of ovarian cancer to improve the post-operative treatment and therefore the survival of these patients. Radioimmunoscintigraphy using monoclonal antibodies against a differentiation antigen (HMFG2) was performed in 25 patients, each of them having an ovarian tumour. The results were satisfactory, thus we decided to scan patients with ovarian cancer routinely as part of other investigations performed in the post-treatment follow-up of these patients. Each patient underwent a second look operation to assess if there was residual disease. Radioimmunoscintigraphy, including Single Photon Emission Computed Tomography (SPECT) was able to detect malignant lesions in some patients which could not be found by other non-invasive investigations. In these patients, the decision on further management was based upon the radioimmunoscintigraphy results. We conclude that radioimmunoscintigraphy, including SPECT, using an appropriate monoclonal antibody such as HMFG2, is able to improve the management of patients with ovarian cancer.

### $^{123}$I-NDOG$_2$ anti-placental alkaline phosphatase imaging of ovarian tumours

E.R. Davies[1], J.O. Davies[2], P.C. Jackson[3], K. Howe[2], E.M. Pitcher[3], C.S. Sadowski[4], G.M. Stirrat[2] & C.A. Sunderland[2]

*Departments of [1]Radiodiagnosis, [2]Obstetrics and Gynaecology, [3]Medical Physics and [4]Pharmacy, United Bristol Hospitals, UK*

The use of radiolabelled monoclonal antibodies offers important opportunities for diagnosing and treating tumours. Confidence in these potential uses requires understanding of the biodistribution of the radiopharmaceutical in tumour-free and tumour-bearing regions. Twenty patients with suspected or surgically confirmed ovarian carcinoma were studied with a monoclonal antibody (NDOG$_2$) labelled with $^{123}$I and directed against placental alkaline phosphatase. Scans of the trunk were done 10 min, 4 h and 20 h after giving 250 $\mu$g of labelled antibody i.v. Changes in the biodistribution of radioactivity were monitored and compared with identical studies in 5 patients with carcinoma of the breast who had tumour free abdomina and pelves. Tumour sites were identified in 16 patients (80%) but the uptake patterns varied because of renal clearance and redistribution of radioactivity from the blood pool to other compartments. Known tumours were not shown in the liver (1), para-aortic lymph nodes (1) and peritoneum seedlings (2). False positives arose because of intestinal activity (2) and subtraction artefact (1). A whole body dose of 16.3 $\mu$Sv MBq$^{-1}$ was calculated. The diagnostic potential of the technique is limited by the variability of PLAP expression in tumours. The agent has not been used for therapy at present.

### Radioimmunolocalisation of the c-*myc* onocogene product in patients with lung cancer

S.Y.T. Chan, A. Ritson & K. Sikora

*Ludwig Institute for Cancer Research, MRC Centre, Cambridge, UK*

We have studied the clinical application of a set of monoclonal antibodies raised by peptide immunisation against fragments of the human c-*myc* oncogene product. One such antibody, Myc-1 CT14, was derived against the 14 amino acid carboxy terminus of the c-*myc*. This mouse monoclonal antibody (an IgG1$\kappa$) was grown in ascites and purified. One milligram of antibody radiolabelled with 1 mCi $^{131}$I was injected i.v. into 10 patients with primary lung cancer and 10 patients with lung metastases from tumours arising in different organs. There was selective uptake of Myc-1 CT14 at the primary tumour site of all 10 patients with carcinoma of the bronchus suggesting a large quantity of the c-*myc* oncoprotein in these areas. Metastases derived from bronchial carcinoma did not take up the antibody. Ten patients with pulmonary metastases arising from sites other than lung also failed to take up Myc-1 CT14. The cellular location of the c-*myc* oncoprotein is known to be nuclear and we conclude that detection in large primary tumours was due to cell death and subsequent release of the protein. Such antibodies may be of use in monitoring tumour load and response to therapy.

### Antibody guided tumour detection using $^{131}$I labelled F(ab′)$_2$ fragments of an anti-CEA moab

P. Riva[1,2], G. Paganelli[1,2], G. Cacciaguerra[1], G. Gentili[1], L. Marri[1], V. Tison[1], M. Marangolo[1] & D. Amadori[1]

*[1]Istituto Oncologico Romagnolo and [2]Servizio di Medicina Nucleare, Ospedale M. Bufalini, Cesena, Italy*

Twenty-eight patients with primary and/or metastatic gastro-intestinal cancer were studied by external scintigraphy after i.v. injection of $^{131}$I-labelled F(ab′)$_2$ fragments of a monoclonal antibody raised against carcinoembryonic antigen (FO23C5 – Sorin Biomedica). Primary tumours were seen in 11 out of 11 patients. Metastases were seen in 60-100% of cases depending on the different organs investigated.

Best images were obtained at 72, 96 and 120 h. Lesions detected were confirmed either by conventional techniques such as X-ray, CT, ultrasound, or by surgical removal. Where possible the *in vivo* results were confirmed *in vitro* by immunoperoxidase staining of surgically removed tissues. As a negative control, patients having positive scans received an equal amount of a non-specific F(ab′)$_2$ fragment (225.28S). No positive scans were obtained in any cases. In order to improve tumour to background ratio, some of these patients were injected i.p. The results are encouraging.

### SESSION 5
(Chairman: D.A.G. Galton)

### Immunotoxins as anti-tumour agents

E.J. Wawrzynczak & P.E. Thorpe

*Imperial Cancer Research Fund, Lincoln's Inn Fields, London, UK*

Conjugates prepared by linking toxins to monoclonal antibodies are cytotoxic to cells bearing the target antigen. The toxins most widely used have been plant proteins such as ricin, from the castor bean, and abrin, from the jequirity bean. Each consists of a B-chain, by which the toxin binds to galactose-containing molecules on cell surfaces and an A-chain, which kills the cells by inactivating ribosomes. It is possible to link the intact toxin to the antibody and block the galactose binding site(s) on the toxin B-chains to prevent the immunotoxin from binding to and killing non-target cells. Alternatively, the A-chain can be attached directly to the anti-tumour antibody via a disulphide bond.

A panel of immunotoxins was prepared from the OX7 antibody against the mouse Thy 1.1 antigen linked to the A-chains of abrin or ricin or to two ribosome-inactivating proteins which act in a similar fashion: Saporin from *Saponaria officinalis* and *Momordica charantia* inhibitors. The *in vitro* cytotoxicity of an immunotoxin did not prove to be a good guide to its anti-tumour effect *in vivo*. Thus, the anti-Thy 1.1-ricin A chain conjugate inhibited protein synthesis in AKR-A lymphoma cells by 50% (ID50) at a concentration of $2.7 \times 10^{-11}$ M. This conjugate extended the median survival time of mice with lymphoma by 9 days, corresponding to the killing of 99.9% of tumour cells. In contrast, the anti-Thy 1.1-saporin conjugate, which had an ID50 of $6.7 \times 10^{-11}$ M, extended the median survival time by 24 days. This corresponds to killing of between 99.99% and 99.999% of AKR-A cells.

### T-lymphocyte depletion of donor bone marrow in human bone marrow transplantation

E. Gordon-Smith

*Department of Haematology, Royal Postgraduate Medical School, London, UK*

That donor lymphocytes, particularly the T-cell subsets, are instrumental in provoking graft *versus* host disease (GVHD) has been documented in many animal models. The original descriptions of GVHD showed that the graft *versus* host response (GVHR) was dependent on immunologically competent lymphocytes in the donor graft, a failure of host lymphocytes to reject the graft and the presence of a major histocompatibility difference between donor and recipient. The severity of the GVHR was proportional to the number of immunocompetent donor cells transferred and to the degree of histocompatibility difference. The GVHR probably involves lymphocytotoxicity directed against host lymphoreticular tissues. GVHD is a consequence of this but is not necessarily dependent on specific donor lymphocytotoxicity directed against the target organ. Host factors are important in the development of the disease. The further elucidation of the GVHR which showed that mature T-lymphocytes were responsible for the reaction and the suspicion that immune reconstitution could occur after bone marrow transplantation from lymphocyte precursors in the donor marrow which would not provoke the GVHR has led to many attempts to remove mature T-lymphocytes from the donor marrow before infusion. The urgency of the attempts to reduce GVHD arose out of the observation that acute GVHD and its complications was the major cause of death related directly to bone marrow transplantation and was the complication which limited the use of transplantation to matched sibling donors.

### SESSION 5 (Open Papers)
(Chairman: D.A.G. Galton)

### Change in binding specificity of an anti-tumour monoclonal antibody after chemical modification

S. Canevari, D. Mezzanzanica, R. Orlandi, M. Ripamonti & M.I. Colnaghi

*Istituto Nazionale Tumori, Milan, Italy*

The use of monoclonal antibodies (MAbs) for *in vivo* therapeutic approaches depends largely on their specificity. During the characterisation of ricin A chain-murine MAb conjugates, we found that the binding specificity of a MAb raised against human ovarian carcinoma (MOv2) seemed altered, whereas its cytotoxic specificity remained unchanged. Therefore, the binding reactivity of unmodified MOv2, conjugate intermediate MOv2-PDP and MOv2-A chain, was tested on 10 different histological types of human tumour cell lines. These three reagents bound with the two reference cell lines were tested (the ovary carcinoma SW626 and the colon carcinoma HT-29). The MOv2-PDP and MOv2-A chain also reacted with 2 cell lines which were unreactive with the unmodified MOv2 (the melanoma MALME 3M and the breast carcinoma MCF-7).

To elucidate the significance of these findings, the following experiments were performed: cross inhibitions between the unmodified and modified MAbs; comparative absorption tests with relevant and apparently irrelevant cell lines; biochemical analysis of the target antigens. The results suggest that after chemical modification the MAb MOv2 increases its binding activity, so that even low numbers of antigenic sites which may be present on apparently irrelevant cell lines can be detected.

**Depression of glioma allografts by antibody-coupled T cells**

Th. Bilzer[1], S. Aumann[1] & D. Stavrou[2]

[1]*Institute of Animal Pathology, University of Munich, and* [2]*Institute of Pathology, Clinicum Bogenhausen, Technical University of Munich, FRG*

Allografts of experimental rat brain glioma in immunodeficient mice were treated with T cells coupled with the monoclonal glioma antibody 14AC1. The antibody has been produced by fusion of splenocytes from glioma-hyperimmunized BALB/c mice and the X63-Ag8.653 mouse myeloma line. The antibody belongs to the IgG2a isotype (Stavrou *et al.*, 1983, *Eur. J. Cancer Clin. Oncol.*, **19**, 1439).

T cells were collected from peripheral blood, spleen and lymph nodes of normal healthy BALB/c donors by depleting IgG-pos cells after Ficoll-Hypaque flotation (Bilzer *et al.*, 1982, *Anticancer Res.*, **2**, 345). T cell-antibody binding was confirmed by micro-ELISA prior to application.

Five million T cells franked with hybridoma ascites were transferred on days 2, 4 6 and 12 after tumour implantation. The mean tumour mass determined in day 28 was 5.9 g, whereas mice treated with the antibody alone revealed 14.3 g on average. Untreated mice as well as mice treated with normal T cells had to be killed between days 14 and 21 because of tumour size and perforation.

**Results of T-cell depletion with CAMPATH-1 in bone marrow transplantation (BMT) for chronic granulocytic leukaemia (CGL)**

J.F. Apperley[1], J.M. Hows[1], G. Hale[2], H. Waldmann[2], E.C. Gordon-Smith[1] & J.M. Goldman[1]

[1]*Department of Haematology, Royal Postgraduate Medical School, London, and* [2]*Department of Pathology, University of Cambridge, UK*

We have transplanted 27 patients with CGL using a rat monoclonal antibody, CAMPATH-1, for T-cell depletion. The patients can be divided into 'good risk' (16 patients in chronic phase (CP)) and 'poor risk' (two in second CP, three in acceleration, two in blast crisis, one with busulphan induced aplasia and three in CP from mismatched family donors). Conditioning consisted of daunorubicin 60 $mg\,m^{-2}$, cyclophosphamide 120 $mg\,kg^{-1}$ and TBI of 10–12 Gy in divided doses. Cyclosporine was given post-BMT to 21 of 27 patients. Donor marrow was treated with CAMPATH-1 *ex vivo*, and donor serum was used as the source of complement. The mean cell dose given was $2.7 \times 10^8\,kg^{-1}$ (range 1.3–4.3) of the 'good risk' group. All are alive with Karnofsky scores of 80–100% at 21–418 days. Engraftment was satisfactory with the average number of days to a neutrophil counts $>0.5 \times 10^9\,l^{-1}$ being 21 (range 11–33). Acute GVHD (Grade I–II) has occurred in only four patients to date, and chronic GVHD in four of the 11 evaluable patients. Of the 'bad risk', four are alive at 109–365 days. Three died of graft failure, 2 of idiopathic interstitial pneumonitis and 2 of CMV pneumonitis. Acute GVHD (Grade II–IV) occurred in 5 patients and chronic GVHD in 2 of the 4 patients surviving beyond 100 days. Graft failure was seen in five patients and, despite further conditioning and a second BMT without T-cell depletion for these patients, satisfactory engraftment has not been achieved in any case to date. We conclude that: (1) T-cell depletion successfully reduces the incidence and severity of GVHD, but (2) the incidence of graft failure in 'poor risk' patients is high, and the chances of achieving successful engraftment with a second BMT are low.

### Sensitivity of malignant lymphoid cells to ricin A-chain immunotoxins

G. Laurent, P. Casellas, J.M. Derocq, P. Poncelet, D. Dussossoy & F. Jansen

*Centre de Recherches Clin-Midy Sanofi, Montpellier, France*

A total of 32 immunotoxins (ITs) have been prepared coupling ricin A-chain with monoclonal antibodies reacting with several differentiation antigens expressed in the cell membrance of various malignant lymphoid cells. Twenty-two anti-T, nine anti-B, and one anti-HAL-DR ITs were evaluated using a protein synthesis inhibition assay. Measuring $^{14}C$-leucine incorporation, the Ligand Enhanced Specific Activity (LESA) factor (Casellas, P. *et al.*, 1982, *Int. J. Cancer*, **30**, 437) has been estimated for each IT. This study showed that the specific activity of ITs can differ from one class of antigen to another, LESA factor varying from 10 to $10^6$ in the presence of activators ($NH_4Cl$ or monensin). Among anti-T ITs, CD5 and CD7 anti-T ITs were found to be the most active (LESA factor varying from $10^2$ to $10^6$) while four CD8 and two CD3 anti-T ITs did not show any activity. Anti-CALLA ITs had poor efficiency. Only two out of nine anti-B ITs showed a certain level of killing efficacy (LESA factor $>10^3$). Furthermore, this study showed that the LESA factor can differ within the same cluster of differentiation up to 4 log amplitude. Finally, this study suggests the influence of both antigens and antibodies on the cell-killing efficacy of ITs.

### Abrin and ricin immunotoxins against melanoma. Toxicity related to binding, internalisation and processing

A. Pihl, A. Godal & Ø. Fodstad

*The Norwegian Institute for Cancer Research, Montebello, Oslo, Norway*

Conjugates of abrin and ricin with 2 antimelanoma antibodies, 9.2.27 and anti-p21o, were purified as described by Thorpe *et al.*, *Eur. J. Biochem.*, 1984, **140**, 63). The abrin immunotoxins were far more specific than the ricin conjugates, which were highly toxic also to a number of non-melanoma tumour lines. The 8 melanoma cell lines studied were 20 to 500 times more sensitive to the abrin than to the corresponding ricin conjugates. The melanoma lines differed over a 250-fold range in their sensitivity to the abrin conjugates. The differences could not be accounted for by different levels of antigen expression, which varied over a 12-fold range, but largely reflected the associated differences, over a 4,000-fold range, in the sensitivity of cells to the native toxins.

Comparisons were carried out with respect to toxicity, binding, internalisation and degradation of labelled conjugate and its moieties in 2 cell lines with different antigen expression. Experiments in which the cells were preincubated with lactose, antibody, or both, suggested that the immunotoxins may be taken up by two different routes and that in cells of different sensitivity to the conjugates, differences in rate of processing were more important than differences in rates of internalisation.

### Use of monodisperse, magnetic particles for removal of B-lymphoma cells from human bone marrow

G. Kvalheim, Ø. Fodstad, K. Nustad, A. Pihl & S. Funderud

*The Norwegian Institute for Cancer Research, Montebello, Oslo, Norway*

Several approaches involving monoclonal antibodies have been used in attempts to remove tumour cells from bone marrow destined for autologous transplantation (Ritz *et al.*, 1982, *Lancet*, **60**, 1266; Muirhead *et al.*, 1983, *Blood*, **62**, 327; Treleaven *et al.*, 1984, *Lancet*, **i**, 70). The success of such procedures will depend critically on the specificity and reactivity of the antibodies used. Here we report on the removal of B-lymphoma cells from human bone marrow using an improved generation of monodisperse magnetic polymer particles prepared by Ugelstad *et al.* and 3 B-Cell specific monoclonal antibodies prepared in this laboratory. The antibodies (AB1, AB2 and HH2) were of the IgM type and were either physically adsorbed or chemically bound to the particles. Four different Burkitt lymphoma cell lines (Rael, Raji, Bjab and Balm-1) were used. The tumour cells were mixed 1:1 with human bone marrow and a 25-fold excess of the charged particles and incubated in RPMI medium at 4°C with frequent turning. After 30 min the magnetic beads were removed by placing the tissue bottles for 1 min on flat cobalt-samarium magnets. The number of remaining tumour cells in the supernatant was determined in a soft agar assay. It was found that the procedure was capable of depleting the tumour cells by up to 5 logs, without serious effect on the marrow progenitor cells, as measured in GM and GEMM assays.

### SESSION 6
(Chairman: A.A. Epenetos)

### Antibody-drug conjugates

M.J. Embleton

*Cancer Research Campaign Laboratories, University of Nottingham, UK*

In order to prepare active anti-cancer drug-antibody conjugates a number of basic requirements must be met. The candidate antibody must react selectively with tumour cells and localise to tumour tissue *in vivo,* and must withstand chemical substitution. The drug should be active against the target cells, and its structure must include chemically reactive sites distinct from functional sites in order to allow attachment without loss of anti-tumour activity. With these requirements fulfilled, there is a limit to the number of drug molecules which can be attached directly to the antibody molecule without irreversibly destroying its ability to bind to antigen but the molar ratio of drug: antibody may be substantially increased by the use of a highly substituted carrier molecule instead of drug alone.

Using the above criteria, conjugates of an anti-human osteosarcoma monoclonal antibody (791T/36) have been prepared with the cytotoxic drugs vindesine, daunomycin and methotrexate, the latter including drug-carrier-antibody conjugates. These conjugates were cytotoxic *in vitro* for tumour target cells which express the 791T/36-defined antigen, but were relatively non-toxic to non-antigenic cells. *In vivo,* radio-labelled conjugates localised to tumour xenografts in immune-deprived mice, and certain conjugates had a significant tumour-suppressive effect at doses containing therapeutic levels of drug. The main advantage of the conjugates was lack of toxicity to the murine hosts at levels greatly exceeding the LD50 of free drug. Drug-antibody conjugates appear to hold promise for future clinical application.

### Dosimetry of radiolabelled antibodies

M.J. Myers & G.R. Hooker

*Departments of Medical Physics and Radiotherapy, Hammersmith Hospital, London, UK*

The accuracy of dosimetric calculations depends on how well the distribution and time course of the therapeutic radionuclide can be defined. A number of studies carried out using different $^{131}I$ labelled antibodies for treatment of the peritoneum, pericardium, pleura and gliomas using intravenous, intra-arterial and intracavitary administration has enabled the kinetics of the labelled antibody *in vivo* to be investigated and measured with a fair accuracy. Problems still exist in accurately localising the radionuclide and determining its volume of distribution even on a macroscopic scale. A comparison has also been carried out between pre- and post-treatment behaviour of the radiolabelled antibody so that a therapeutic regime can be predicted from a study with much lower doses.

Examples of dosimetric studies, explaining the methods of obtaining the data and the calculations involved, both before and during therapy and over the range of treatments and types of administration, will be presented and the problems in calculating the doses to the tumour and the rest of the body will be discussed.

### Clinical results with regional antibody-guided irradiation

A.A. Epenetos

*On behalf of Hammersmith Oncology Group, Royal Postgraduate Medical School and Hammersmith Hospital, and Imperial Cancer Research Fund, London, UK*

Recently we described a new therapeutic method termed regional antibody guided irradiation where, instead of intravenously, antibodies were administered regionally into body cavities for treatment of regionally confined tumours.

Fifteen patients with ovarian cancer were treated intraperitoneally with $^{131}I$-radiolabelled antibodies HMFG1, HMFG2, AUA1, H17E2 given singly or as a mixture. Toxicity (reversible diarrhoea, leucopenia and thrombocytopenia) was noted at higher than 100 mCi activities. Most patients benefited symptomatically and, furthermore, in 6 out of 9 patients with stage III and minimal residual disease, a complete remission was achieved and maintained for 3–18 months after treatment.

Antibodies were introduced intracavitary for the treatment of malignant pleural effusions (20–100 mCi $^{131}I$). There has been resolution with no evidence of recurrence in 6 out of 7 pleural and 3 out of 3 pericardial effucions. Two patients with Grade IV glioma of brain unresponsive to other therapies were treated by arterial infusions of radio-labelled antibodies (A9, EGFR1) 45–102 mCi $^{131}I$). A sustained clinical improvement was noted.

In conclusion, regionally administered radio-labelled antibodies (A9, EGFR1) (45–102 mCi $^{131}I$). A sustained clinical improvement was noted. survival of some patients with malignant disease.

### SESSION 6 (Open Papers)

(Chairman: A.A. Epenetos)

### Human monoclonal antibodies used in the study of immune reponse in patients with cancer

P.B. Christensen, K. Erb, J.C. Jensenius & B. Nielsen

*Biomedical Laboratory, Institute of Surgery, and Medical Microbiology and Pathology Departments, University of Odense, Denmark*

Human–human hybridoma technology has been used in an attempt to study the humoral immune reactions of colorectal cancer patients against their tumours and the possible reactions also against tumours from other patients.

Seven fusions have been done with lymphocytes from lymph nodes from patients with colorectal cancer, using the human B-lymphoma cell line LICR-LON-HMy-2 as fusion partner. A total of 301 hybrids were obtained. Of these 27 react in enzyme-linked-immuno-sorbent assay (ELISA) with one or more colon cancer cell lines. One of these hybridomas produces antibody which in immunocytochemistry reacts with a panel of colon cancer cell lines and melanoma cell lines but not with normal human lymphocytes. When using immunohistochemistry we found that this antibody in addition reacts with antigen present on autologous and several allogenous colorectal cancers. The study suggests that some patients generate an immune response against their own malignant cells and that the antigens also are expressed by tumour cells from other patients.

### Validity of non-invasive dosimetry for monoclonal antibodies in humans

Nancy D. Hammond, P.J. Moldofsky, R.E. Exten, R.A. Gatenby & G.J. Broder

*Fox Chase Cancer Centre and Jeanes Hospital, Philadelphia, PA, USA*

Two non-invasive methods for quantitation of absolute activity and determination of biological clearance of Iodine-131 labelled $F(ab')_2$ fragments of mouse monoclonal antibody localised in lesions and normal tissue by patients with metastatic colon carcinoma have been evaluated. The quantitation includes kinetic measurements of $^{131}I$ activity in lesions, liver and total body, calculation of absolute activity in lesions, liver and total body and the resultant computation of dose (rads) to these tissues. Absolute activity calculations were based on computer-acquired conjugate views of the organs of interest and also on a 'first pass' approximation of activity administered to the patients. Measurements of patient attenuation were individually determined from $^{131}I$ transmission scans. Patient tumour and liver volume and thickness were calculated from contiguous CT scan sections. For patients analysed, mean tumour and liver volumes were 175.8 and 2018.4 $cm^3$ respectively with the patient attenuation coefficients ranging from 0.088–0.127 $cm^{-1}$. The ratio of tumour to liver activity reached a maximum of 6.1 at 72 h with tumour uptake $\pm 0.01\%$ of administered dose $cm^{-3}$. Tumour doses of $\sim 1$ rad $mCi^{-1}$ administered were found for a typical lesion of 5 cm diameter. The dose to the thyroid gland was calculated for 2 patient populations; one receiving Lugol's solution only at the time of $^{131}I$ administration and the other receiving Lugol's for 3 days prior to $^{131}I$ administration. No significant difference in absorbed dose to the thyroid was found. Lastly, $<10\%$ error was found in utilising these quantitative methods when compared to measurements made with a tissue equivalent phantom. Comparison was also made to actual well-counter assayed samples of malignant and normal tissues obtained from CT-guided needle biopsies or surgical specimens.

### Cytotoxic monoclonal antibodies with tumour specificity as immunotherapeutic agents for metastatic G-I tract adenocarcinoma

J.Y. Douillard, J. Vignoud, C. Maurel & H. Koprowski

*U.211 INSERM, UER Médecine, Nantes, France*

A cytotoxic monoclonal antibody of $IgG_2a$ subclass, namely 17 1A, was used as immunotherapeutic agent in patients presenting with widespread metastatic disease. Twenty patients were infused with 500 mg 17 1A pre-incubated with autologous peripheral blood leucocytes obtained at leucophoresis. No serious side-effects were noticed except in one patient receiving a second antibody infusion. Ten patients got no clinical benefit from 17 1A immunotherapy and died of disease progression $7 \pm 4$ months after therapy. In the remaining 10 cases, disease evolution was impaired after antibody therapy: 5 patients were stabilised and 5 patients showed a decrease in measurable tumour size after treatment. Mean follow-up for these 10 patients is presently $12.5 \pm 5$ months with 9 out of 10 still alive and in good general condition.

This initial therapeutic protocol seems to show some therapeutic activity but should be confirmed by larger studies.

### Antibody-guided radiolocalisation and therapy for neoplastic meningitis

H.B. Coakham, R.B. Richardson, S. Bourne, A.G. Davies & J.T. Kemshead

*Departments of Neurosurgery and Physics, Frenchay Hospital, Bristol, and ICRF Oncology Laboratory, Institute for Child Health, London, UK*

The problems of accessibility of antibody cells that may interfere with efficient targeting against solid tumours can be overcome by local infusion of radiolabelled antibody in certain anatomical sites (Epenetos *et al.*, 1984, *Lancet*, **ii**, 1441).

We report the first use of a radiolabelled monoclonal antibody (MCA) to localise and treat neoplastic meningitis due to a pineal tumour. MCA UJ181.4 which recognises an oncofoetal neuroblast antigen was labelled with $^{131}I$ and infused into the CSF with $^{125}I$-HMFG2 as control. The results of immunoscintigraphy and radiolabelled antibody kinetics in the CSF, blood and urine confirmed specific localisation of the antitumour MCA, with ratios of up to 30:1 specific/non-specific MCA being obtained. On the basis of this data a therapy dose of 870 MBq $^{131}I$ UJ181.4 was given intrathecally. This produced a marked clinical improvement and the patient remains in remission. Our results suggest that direct access of antibody to tumour cells in the CSF pathways is likely to give superior results compared with intravenously injected antibody for solid brain tumours.

### The use of second antibody to accelerate the clearance of therapeutic doses of $^{131}I$ labelled goat anti-CEA in patients

J.A. Ledermann, R.H.J. Begent, F. Searle, A. Green, K.D. Bagshawe, M.G. Glaser & R.G. Dale

*CRC Laboratories, Charing Cross Hospital, London, UK*

In radiommunolocalisation, administration of free second antibody directed against the antitumour antibody accelerates the clearance of the latter from the circulation and increases the tumour:blood ratio. In radioimmunotherapy, higher doses of antibody and radionuclide are used. The purpose of this study was to investigate the effectiveness and safety of second antibody administation for radioimmunotherapy. Patients with unresectable colorectal carcinoma received ~50 mCi of $^{131}I$ conjugated to a polyclonal goat anti-CEA antibody. The second antibody (horse or donkey anti-goat) was given in 2.5–5 times the amount of the first antibody 24 h later and the kinetics of the clearance showed that the administration of the second antibody was associated with a rapid increase in the removal of antibody from the circulation and the body. In one patient the circulating level of $^{131}I$ labelled antibody fell from 40% of its initial value to 8% within 3 h of the administration of the second antibody. By 60 h <10% of the radioactivity remained in the body. The tumour was localised using a gamma camera and the radioactivity in a biopsy specimen of the tumour was four times as great as the radioactivity in the blood.

Therapeutic doses of radiolabelled antibodies given to patients have been well tolerated. The rapid clearance of radioactivity from the body using the second antibody may allow higher doses of radioactivity to be used safely and further studies are now justified.

### Demonstration of the presence of functional HMFG2 antibody and its specific determinant in metastatic breast tumours

A.B. Griffiths[1], N. Padmanabhan[2], C. Lazarus[2], P.S. Sheppard[2] & J. Taylor-Papadimitriou[1]

[1]*Imperial Cancer Research Fund, London, and*
[2]*Guy's Hospital, London, UK*

The aims of the study were to image metastatic breast lesions using radioiodinated tumour associated monoclonal antibody HMFG2 whilst gathering information about antigen expression and antibody behaviour *in vivo*. Imaging was unsuccessful.

The antibody was shown to retain its binding properties after iodination (either $^{123}I$ or $^{131}I$). Circulating labelled antibody was shown to bind to immunoblots of PAGE separated T47D cells in an ELISA assay and by autoradiographs of the immunoblots, a more sensitive assay. Up to 80% of circulating radioactivity could be recovered on immunoglobulin. Autoradiographs of PAGE separated pleural effusion fluid and pleural effusion cell extracts demonstrated the presence of labelled intact HMFG2 antibody in the pleural effusion fluid and also on pleural effusion cells.

Autoradiographs of PAGE separated immunoblots of extracted cutaneous metastases demonstrated intact labelled HMFG2 in the lesion. Circulating HMFG2 antibody could be demonstrated, to bind to its specific determinant on PAGE separated immunoblots of the cutaneous metastatic tumour in both an ELISA assay and autoradiography of the immunoblots.

### Abstracts of Poster Exhibits

### A new sensitive miniature enzyme immunoassay using β-galactosidase/anti β-galactosidase complexes (GAG) for the analysis of solid phase bound antigens

H. Durbin, E. Milligan & W.F. Bodmer

*Imperial Cancer Research Fund, London, UK*

The use of ELISA in laboratory medicine and research is now widespread. However, for situations where target antigen or volume of test solution is limited, the deficiency in existing systems of lack of adequate sensitivity has led to the development of a new extra sensitive micro ELISA.

Monoclonal antibodies to β-galactosidase have been made allowing the use of complexes in a version of the unlabelled enzyme–antienzyme system.

Test volumes of 5 or 10 μl are incubated for 1 h with antigen bound to 60 well Terasaki plates; this may be whole cells $10^3$/well. Two subsequent 1 h incubations, (a) with an antimouse bridging antiserum, and (b) with GAG complexes are followed by a 30 min incubation with a fluorogenic substrate. Fluorescence is detected and recorded on a Leitz MPV Compact MT inverted microscope linked to a HP85 bench computer which controls the automatic scanning process and print-out. A plate is read in one minute and lower levels of $10^{-6}$ M of the substrate reaction product may be detected. Alternatively, quantitative fluorescence may simply be recorded by photography over UV light. By combining the use of a monoclonal anti β-galactosidase/β-galactosidase complex with other refinements such as automation, all-over improvements, not least in sensitivity, have been achieved.

### The expression of placental alkaline phosphatase by normal and gynaecological malignancy tissue defined by the $NDOG_2$ monoclonal antibody

J.O. Davies, K. Howe & G.M. Stirrat

*Department of Obstetrics and Gynaecology, Bristol University, UK*

A murine monoclonal antibody, designated $NDOG_2$, has been developed which recognises the three common allelic forms of placental alkaline phosphatase (PLAP). This antibody has been used in an indirect immunoperoxidase technique to demonstrate PLAP in normal fallopian tube, endocervical and endometrial epithelia as well as normal lung and thymus. Variable degrees of reactive staining with $NDOG_2$ have been found in 64% of 56 cystadenocarcinomas and in 25% of the 44 cystadenomas studied. In addition, 65% of the 17 endometrial carcinomas and 42% of 12 cervical cancers also express this antigen but none of the 3 uterine sarcomas or 3 vulval carcinomas showed any degree of staining.

### The use of a serum assay, based on the $NDOG_2$ monoclonal antibody, to predict the course of the disease in patients with ovarian cancer

J.O. Davies, K.Howe, B.Randle, C.A. Sunderland & G.M. Stirrat

*Department of Obstetrics and Gynaecology, Bristol University, UK*

The $NDOG_2$ monoclonal antibody, which reacts with the common forms of placental alkaline phosphatase (PLAP), has been used to demonstrate reactive staining in 65% of 56 ovarian cancers, predominantly serous cystadenocarcinomas (Sunderland *et al.*, 1984, *Cancer Res.*, **44**, 4496).

To assess the usefulness of this potential tumour marker, $NDOG_2$ has been used as the basis of two serum assays. Assay 1 measures PLAP activity whereas Assay 2 measures the NDOG determinant.

There was a close correlation between positive reactive staining with $NDOG_2$ and pre-operative serum levels using both assays. Assay 1 proved to be of predictive value in 12 out of 44 patients studied and Assay 2 was of value, both in these cases and in a further 8 patients. In an additional 7 cases, Assay 2 initially predicted the course of the disease but subsequently fell, inappropriately, prior to the patient's death.

Assay 2 offers potential in the management of patients with ovarian cancer, particularly those with serous cystadenocarcinomas. However, as $NDOG_2$ recognises a tumour associated, as opposed to a tumour specific, antigen, care should be taken in interpreting the significance of a single elevated result.

### The use of monoclonal antibodies to *ras*-oncogene product in the diagnosis of carcinoma of the colon and rectum

N. Habib[1], H. Niman[2], A. Thompson[1], D. Kersten[1] & C. Wood[1]

[1]*Department of Surgery, Royal Postgraduate Medical School, London, UK and* [2]*Department of Molecular Biology, Scripps Institute, La Jolla, California, USA*

The expression of c-*ras* oncogene has been demonstrated in carcinoma of the colon and rectum. The activated cellular *ras*-oncogene synthesizes a protein (p21) with a point mutation at position 12. We developed monoclonal antibodies against amino acid sequences of p21 centred on this specific point mutation.

Using the peroxidase-anti-peroxidase techniques we stained sections from colorectal cancer using monoclonal antibodies to p21. Staining was positive in all tumour sections of 8 patients with colorectal malignancies and negative in both proximal and distal resection margins of colectomy specimens in these patients. Monoclonal antibody binding was found in both nucleus and inner side of the cell membrane.

Staining of colorectal tumour tissue with monoclonal antibodies against *ras*-oncogene products may open new avenues for immunohistological diagnosis.

### Uses of monoclonal antibodies specific for the human oncoprotein $p62^{c\text{-}myc}$ in molecular biological and clinical analyses

G.I. Evan, K. Sikora, J. Stewart, J. Watson & R. Buick

*Ludwig Institute for Cancer Research and MRC Clinical Oncology Unit, MRC Centre, Cambridge, UK*

We have prepared monoclonal antibodies specific for human c-*myc* oncoprotein $p62^{c\text{-}myc}$ by immunisation with synthetic peptides. These antibodies have proved of immense value in analysis of the cell biology of the c-*myc* oncoprotein, demonstrating the protein's subcellular location and properties.

We have also used these antibodies in immunohistological analyses of normal and neoplastic tissues, and in flow cytometric analyses of archival histological material. Our data point to the future uses of these reagents in diagnosis and prognostic assessments from clinical specimens.

### Detection of the c-*myc* oncogene product in colonic polyps and carcinomas

J. Stewart, G. Evan, J. Watson & K. Sikora

*MRC Clinical Oncology Unit and Ludwig Institute for Cancer Research, MRC Centre, Cambridge, UK*

The c-*myc* oncogene has been implicated in the processes of normal cell proliferation and differentiation. Elevated levels of c-*myc* mRNA and its gene product ($p62^{c\text{-}myc}$), have been detected in a variety of solid tumours and cultured cell lines. Its precise role in normal cell function and in neoplastic transformation and progression has yet to be elucidated. We have used a monoclonal antibody, raised by peptide immunisation, to determine the distribution by immunoperoxidase staining of the c-*myc* oncogene product in archival specimens of colonic polyps and carcinomas. Samples from 42 patients with colon carcinoma, 24 with benign polyps and 15 normal colon biopsies were examined. Normal colon revealed maximum staining in the mid-zone of the crypts, corresponding to the zone of differentiation and maturation. The staining was predominantly cytoplasmic. Adenomatous polyps revealed the most intense pattern of staining in areas of dysplastic change. Colonic tumours showed a wide range of staining. Well differentiated tumours contained more cytoplasmic $p62^{c\text{-}myc}$ than poorly differentiated tumours. These findings suggest that the c-*myc* oncogene product may play an important role in the evolution of colonic neoplasia.

**Demonstration of placental alkaline phosphatase (PLAP) and tissue non-specific alkaline phosphatase (AP) in normal lung tissue**

D.E. Pollet, E.J. Nouwen, M.W. Eerdekens, T.W. Briers & M.E. De Broe

*Department of Nephrology-Hypertension, University Hospital of Antwerp, Edegem, Belgium*

PLAP was assessed by an enzyme immunoassay as reported by Pollet *et al.*, 1985, *Clin. Chem.*, **31**, 41. AP was quantified by a similar assay system including an AP specific monoclonal antibody (AP230). Tissue samples were obtained post mortem after donor nephrectomy, at pneumectomy or at the occasion of an induced abortion. The presence of Nagao-type PLAP was assessed by L-Leucine inhibition (1 mmol $l^{-1}$).

Adult lung tissue contained a mean PLAP activity of 78 mU $g^{-1}$ (range 3–181); this is 6.4% (range 1–22) of the total alkaline phosphatase activity. No L-Leucine sensitive PLAP activity could be found in these extracts. The main alkaline phosphatase found in adult lung tissue was tissue non-specific AP as could be demonstrated by the sensitivity to amino acids, L-p-bromotetramisole, neuraminidase treatment and heat inactivation analysis. Foetal lung tissue ($n=5$) between 11 and 15 weeks of gestation did not contain a substantial amount (>2 U $kg^{-1}$) of PLAP. The biochemical results were confirmed by immunohistological localisation of PLAP on the respiratory bronchioli and alveoli, and by the histological localisation of AP in the tracheal and bronchial epithelium, the tracheal and bronchiolar glands, and the endothelium of small blood vessels.

**Serum levels of hPLAP and CA 125 in benign and malignant disease and immunohistochemical evidence for CA 125 hepatic uptake**

M.W. Eerdekens, E.J. Nouwen, D.E. Pollet, T.W. Briers & M.E. De Broe

*Department of Nephrology-Hypertension, University Hospital of Antwerp, Edegem, Belgium*

In a comparative study, human placental alkaline phosphatase (hPLAP), CA 125 and CEA were determined in sera of patients with ovarian (20) and non-ovarian tumours (45), and in patients with non-malignant disorders (126), using monoclonal antibodies against these antigens. The latter group consisted of 10 diabetic, 19 renal insufficiency, 50 chronic dialysis, 24 icteric non-cirrhotic, 9 icteric cirrhotic and 14 cirrhotic nonicteric patients. hPLAP and CA 125 had the same sensitivity (20%) for non-ovarian tumours, whereas that of CEA was 45%. In ovarian cancer, the sensitivity was 45% for hPLAP, 69% for CA 125 and only 10% for CEA. hPLAP had the lowest false-positivity (2%) in patients with non-malignant disorders (1 diabetic and 2 patients on chronic dialysis). In contrast, CA 125 and CEA were increased in 23% and 18%, respectively, of patients with benign pathologies. In this group, cirrhotic patients had the highest prevalence (88%) of elevated serum CA 125 levels. CA 125 was localised immunohistochemically in normal liver tissue of a patient with a metastatic poorly differentiated pancreatic adenocarcinoma having increased serum levels of hPLAP, CEA and CA 125. Positive CA 125 staining was observed intracellularly in hepatocytic lysosomes and was absent in Kuppfer cells. Normal liver does not contain detectable CA 125 staining. This observation is suggestive for uptake of CA 125 by the hepatocytic asialoglycoprotein carrier mechanism. This hypothesis also explains the elevated serum levels of CA 125 in patients with impaired liver function. The sensitivity of hPLAP in detecting ovarian cancer is slightly inferior to that of CA 125, but its specificity is much higher.

**Monitoring advanced seropapillary anaplastic ovarian cancer by determinations of CA 125**

T. Högberg[1] & A. Hedin[2]

[1]*Department of Gynaecological Oncology, University Hospital, Linköping, Sweden and* [2]*Pharmacia AB, Uppsala, Sweden*

The clinical value of CA 125 as a marker in advanced ovarian cancer during and after therapy was studied.

In 26 patients with seropapillary or anaplastic (FIGO histological group 1c or 5) ovarian cancer FIGO stage III and IV, serum samples were drawn and then frozen for later analyses before each chemotherapy cycle, before second look operations and at each visit during follow up. First sample was taken at different phases of disease. Analyses were carried out with kits from International CIS, France.

Fifteen of 26 patients (60%) showed positive marker ($>35\,U\,ml^{-1}$) at any time. If patients clinically not expected to show high marker are excluded, 15/20 (75%) showed positive marker. There were definite patterns over time depicting tumour response during chemotherapy. There was a correlation between pre-operative marker value and findings during second look operations, although there could be widespread cancer with a normal marker value. In two cases relapsing during follow up there was a lead time of at least 6.8 and 1 month respectively between high marker value and clinically evident tumour.

It is concluded that CA 125 correlates well with evolution of disease in ~80% of the cases; could perhaps be used as an early sign of sensitivity to cytostatic drugs; correlates with findings at second look, but cannot be used as a subsitute for operation, and can signal a relapse before it becomes clinically evident.

**Human anti-mouse immunoglobulin responses in patients receiving monoclonal antibodies**

N.S. Courtenay-Luck, S. Needham, R. Moore, M. Larche, B. Dhokia, M. Ritter & A.A. Epenetos

*Departments of Immunology and Radiotherapy and Oncology, Royal Postgraduate Medical School, London, UK*

The use of mouse monoclonal antibodies in the diagnosis and therapy of malignant neoplasms is proving to be of great clinical interest. A consequence of using mouse antibodies is stimulation of a vast array of immunological responses including a human-anti-mouse response.

Experiments carried out using patients' sera after both a diagnostic and therapeutic injection of monoclonal antibodies labelled with radioactive iodine show quite clearly the presence of human anti-mouse antibodies.

Screening of our patients' sera has been done by employing two methods: (1) a modified enzyme-linked immunosorbent assay, ELISA, and (2) hybridoma targeting which employs the use of antibody bearing hybridoma cell cytospins.

Our studies show that the reponse is anti-mouse not anti-idiotypic and the response is greatest if the interval between the first and second injections is greater than 10 days, due to the mainly IgG secondary immune response, and negligible if patients are given the diagnostic and therapeutic injections within 7 days of each other, hence preventing the development of a large secondary immune response.

**Study of the expression of the HLA-DR antigen on cloned melanoma cell lines with a potent monoclonal antibody**

S. Hallez, F. Vessière, C. Lemoine, K. Willard, T. Boon & P. De Meyts

*ICP, Unite HORM 7524, and LICR, 75 and 74 av. Hippocrate, Brussels, Belgium*

We have produced a monoclonal antibody against the class II MHC HLA-DR antigen. $^{125}$I-antibody 03-D7 binds irreversibly to the surface of DR+ lymphoblastoid cell lines. It binds to all DR+ cell lines independently of haplotype. Scatchard plots show an apparent affinity of more than $10^{10}\,M^{-1}$. The antibody binds in Western blots to the purified $\beta$ chain HLA-DR. We used the monoclonal 03-D7 to investigate its binding to twelve different cloned melanoma cell lines. Three cell lines did not show binding of the $^{125}$I-monoclonal, while the nine others showed binding with a great variation in the number ($3.4\times10^5$ to $1.17\times10^7$ sites/cell) and affinity ($2.9\times10^8$ to $1.6\times10^{10}\,M^{-1}$). This suggests that the modality of expression of DR molecules on the surface of melanoma cells may greatly vary. The labelled monoclonal may be a useful tool in positive identification of melanoma cells.

**Immunohistochemical studies of mammary carcinoma with the monoclonal antibody MC 211**

C. Maurel, G. Aillet, M. Kremer, J.F. Chatal & J.Y. Douillard

*U.211 INSERM, UER Médecine, Nantes, France*

Monoclonal antibody MC211 (IgA class) was produced by hybridomas after immunisation of BALB-c mice with membrane-enriched fractions of human mammary carcinoma and breast cell line MCF7. Using an immunohistochemical method, with the avidin-biotin peroxidase complex, the reactivity of MC211 MoAb was studied in formalin-fixed paraffin embedded sections of surgical specimens.

Twenty-five of the 27 mammary adenocarcinomas tested were strongly positive (50 to 80% positive cells); the 2 others were weakly positive (10% positive cells); one mammary endocrine carcinoma was negative; non-breast adenocarcinomas were tested; digestive carcinomas were positive (7/7: colon, pancreas, stomach adenocarcinomas; 3/4 ovarian carcinomas were weakly positive; the two pulmonary adenocarcinomas tested were weakly positive;

The other tumours tested were negative: 3 endocrine tumours; 2 pleural mesotheliomas; 2 malignant melanomas; 3 sarcomas.

Therefore, MC211 monoclonal antibody seems to be of interest in the diagnosis of adenocarcinomas and will be further evaluated in immunohistochemistry for the detection of bone marrow micrometastases from breast carcinomas at the time of surgery and as radioimmunoimaging agent for the diagnosis of lymph node involvement and other metastases.

**Monoclonal antibodies to liver F-antigen discriminate between parenchymal and ductular epithelial cells in adult human hepatic tissue**

P.J. Higgins

*Memorial Sloan-Kettering Cancer Center, New York, USA*

Hybridoma cell lines producing monoclonal antibodies (MAbs) to F-antigen of liver were prepared by fusion of NS-1 myeloma cells with spleen cells obtained from CBA mice immunised with saline extracts of BALB/c mouse liver. MAbs reactive with F-antigen were selected by comparative immuno-blot screening using electrophoretically separated proteins from BALB/c liver and polyclonal anti-F-antigen sera. Immunohistochemical staining of paraffin-embedded and cleared sections of normal adult human liver with two such MAbs (I/2, I/5) defined epitopes of F-antigen expressed only on lobular parenchymal hepatocytes; bile duct epithelium, sinusoidal lining cells, endothelial tissue and portal tract mesenchymal elements were unreactive with these MAbs. In a screen of 50 different malignant and benign hepatic neoplasms of both murine and human origin, MAbs I/2 and I/5 clearly discriminated hepatocellular from ductular carcinomas and adenomatous foci from bile duct hyperplasia. It appeared that protease pre-treatment of liver sections was necessary for maximum immunocytochemical resolution of F-antigen with I/2 and I/5. Work is currently in progress to define, using the current panel of MAbs, the controversial origin of the 'oval' epithelial cell induced in the livers of carcinogen-treated animals.

**Monoclonal antibodies to prostatic specific antigens**

F. Donn[1], W. Becker[2], H. Becker[1], T. Bruns[1], G. Griessonn[1] & A. Passarge[1]

[1]*Urologische Universitatsklinik, Hamburg, and*
[2]*Forschungsinstitut Borstel, FRG*

In order to understand better the mechanism involved in normal and abnormal prostatic growth, we attempted to assess human specific antigens defined by monoclonal antibodies. Balb/c mice were immunised with membrane antigens hyperplasia (BPH) and with protein from rat ventral prostate cytosol that binds estramustine. This antigen was obtained from Leo Research Laboratories and was purified to homogeneity using chromatography on DEAE-cellulose, Sephadex G-100, Octyl-Sepharose CL-4B and polyacrylamide gel electrophoresis. The screening method for the hybridoma supernatants used immunoperoxidase stained frozen sections of human tissues as well as the dot blotting method. The antigens found by this method are of 3 classes: a specific secretion product of the prostate which is polyepithelial and stroma specific. The molecular analysis was defined by $^{35}$S-methionine labelling experiments of cultured prostatic cells and subsequent SDS PAGE after immunoprecipitation of supernatant by the selected hybridoma antibodies. Except for the estramustine binding protein, the functions of the antigens are still unknown and are under investigation. They may be of value in understanding the physiology of normal prostate, benign hyperplasia and cancer.

### Cross reactivity of the antimelanoma antibody 9.2.27 with human sarcomas and normal fibroblasts

A. Godal. Ø. Bruland, Ø. Fodstad, A.C. Morgan & A. Pihl

*The Norwegian Institute for Cancer Research, Montebello, Oslo, Norway*

The antibody 9.2.27 directed against the 250 K melanoma-associated antigen (Morgan *et al.*, 1981, *Hybridoma*, **1**, 27) has been extensively studied. Although the antigen is present on some skin cells (Ross *et al.*, 1984, *Cancer Res.*, **44**, 4642), it has not been identified on other tumour types and has been considered specific for melanomas. More than 90% of human melanomas express the antigen (Oldham *et al.*, 1984, *J. Clin. Oncol.*, **2**, 1235), and recently the application of the 9.2.27 antibody to the diagnosis and therapy of melanomas has been intensively explored.

During a study of antisarcoma antibodies the accidental observation was made that the 9.2.27 antibody binds strongly to sarcoma cells. Binding to single cell suspensions was demonstrated by indirect immunofluorescence and by radioactivity measurements using labelled antibody, and binding to cryostat sections was shown by peroxidase staining. *In vivo* binding of radiolabelled antibody to sarcomas was demonstrated in tumour bearing athymic mice as well as in patients with metastaic sarcoma. Binding of 9.2.27 also occurs to proliferating fibroblasts in primary cultures. Immunoprecipitation studies demonstrated that the antigen on fibroblasts and sarcoma cells is identical to that present on melanoma cells.

### Potential application of a new monoclonal antibody, highly specific for human sarcomas

Ø. Bruland, Ø. Fodstad, S. Funderud & A. Pihl

*The Norwegian Institute for Cancer Research, Montebello, Oslo, Norway*

The sarcomas represent a heterogeneous group of tumours with multiple subtypes that are difficult to characterise in detail. Recently, we have succeeded in preparing a monoclonal antibody which is highly specific for human sarcomas. Cells from a xenografted human osteosarcoma were used for immunisation and antibody-secreting hybridomas were screened by indirect fluorescence using unfixed cells. Fibroblasts from several sarcoma patients were used as negative controls. The new antibody, TP-1 (isotype Ig2a), recognises a trypsin-resistant protein of MW 105,000. When tested on acetone-fixed cryostat sections, using a 3-step peroxidase method, the antibody showed no significant binding to a wide range of normal adult tissues, but bound weakly to foetal kidney tubules. Forty-one non-sarcoma malignancies of different histological types were negative. In 29 sarcomas tested, positive staining was obtained in 6/6 osteosarcomas, 5/5 malignant fibrous histiocytomas, 1/1 chondrosarcoma, 2/2 synovial sarcomas, 2/2 malignant hemangiopericytomas and 3/5 unclassified sarcomas. Three rhabdomyo-, 2 leiomyo- and 3 lipo-sarcomas were negative. The specificity profile renders TP-1 a potentially useful reagent in the histological identification and subclassification of sarcomas. Using a radiolabelled $F(ab')_2$ fragment we have successfully imaged a human sarcoma growing s.c. in nude mice.

### Rat monoclonal antibodies to human uveal melanomas

B.E. Damato[1,2], W.S. Foulds[2] & A. Campbell[1]

*Departments of [1]Biochemistry and [2]Ophthalmology, University of Glasgow, UK*

Previous investigations into the immunology of uveal melanomas have been limited by the lack of well defined antigens.

Rat monoclonal antibodies to uveal melanomas have been produced using fresh unfixed tissue. A panel of these antibodies is being used to determine the antigenic profiles of a bank of human uveal melanomas by means of ELISA. Considerable heterogeneity amongst primary tumours has been revealed.

The antigenic profile of each tumour is being correlated with the tumour cell type and patient survival. It is envisaged that these studies will enable clinically significant antigens to be identified.

### Monocyte maturation index as a non-specific tumour marker in cancer patients

Z. Rudolf, G. Sersa & G. Krosl

*The Institute of Oncology, Ljubljana, Yugoslavia*

The maturation index was compared with pertinent clinical data (stage of the disease, course of the disease, and response to treatment) in order to establish clinical significance. The influence of autologous sera was tested in cancer patients as well as in experimental models (peritoneal macrophages from CBA mice). Monocytes from peripheral blood were cultured in both autologous and newborn-calf serum. Maturation index was expressed as the percentage of monocytes placed in culture which were present as adherent macrophages after 48 h. Phagocytic capacity of CBA mouse peritoneal macrophages was expressed as a ratio of activity between cells cultured in patient and healthy donors' sera. Maturation index (MI) was in correlation with the stage of the disease in malignant melanoma patients. The difference between stage I and stage IV patients was significant (8.2% *vs* 2.2%, $P=0.01$). Similarly difference was established between operated and inoperable colorectal cancer patients (21.4% *vs* 5.1%, $P=0.01$). The difference between MI in malignant melanoma patients with complete response and progression of the disease was significant as well (11.7% *vs* 2.2%, $P=0.001$). Autologous sera inhibited the maturation process *in vitro* in cancer patients. In an experimental system autologous sera stimulated the phagocytic capacity of mouse peritoneal macrophages when compared with phagocytic of macrophages cultured in the sera of healthy donors. The correlation between *in vitro* monocyte maturation and clinical factors leads to the conclusion the MI reflects the *in vivo* process, and may prove useful as a marker of tumour load or spread; it may be a sensitive monitor of the effect of treatment, at least in the studied group of patients.

### Tumour heterogeneity and specificity of McAbs – permanent challenges for *in vivo* application

K.S. Zänker & T. Lederer

*Institute for Experimental Surgery, Technical University of Munich, FRG*

Even though the majority of tumours may be monoclonal in origin, a 'clone is not forever' and both phenotypic and genetic mechanisms create extensive diversity within tumour cell populations. In order to evaluate the advantage of specific immune reactions of McAbs, we recently compared McAb (Lederer, 1984, *Br. J. Cancer*, **50**, 567) with naturally occurring polyclonals concerning complement-dependent cytotoxity and possible shift of cycling cells to quiescence. Enlarging our studies, polyclonals from two breast cancer patients were purified (Armour Pharma, FRG) and tested for complement-dependent cytotoxicity against two allogenic human breast cancer cell lines. In comparison, we focussed on a McAb, MGWe 15, recognising an antigen of epithelial cells of cancerous mammary gland. Interestingly, the naturally occurring polyclonals showed complement-dependendent cytotoxicity up to 22% *in vitro*, the McAb failed. Both immunologic probes shifted the human breast cancer into a dormant stage and affected the transmembranal $^{75}$Se-methionine traffic. *In vitro* incubation of the polyclonals with peripheral lymphocytes suggested an affinity directed against suppressorgeneic determinants (OKT8), when monitored by double fluorescence technique, using the Ortho-System®. The McAb was highly specific for the epithelial cells. In conclusion, under some instances, raised McAbs might be 'super specific', without cytotoxic effector function; however, to use such McAbs as specific carriers for tumour toxic agents is an enormous challenge.

### Immunohistochemical studies of primary lung cancer with HMFG1 and HMFG2 monoclonal antibodies

D.V. Skarlos, G. Tiniakos, N. Kalogeropoulos, N. Papacharalambus, N. Nacopoulou, A.A. Epenetos, & J. Taylor-Papadimitriou

*Histopathology Department of NIMTS Hospital and Histopathology Department of Medical School of University of Athens, Greece, Imperial Cancer Research Fund and Royal Postgraduate Medical School, Hammersmith Hospital, London, UK*

Monoclonal antibodies HMFG1 and HMFG2 (Taylor-Papadimitriou, *et al.*, 1981, *Int. J. Cancer*, **28**, 17) raised against milk fat globule membranes react with a broad spectrum of epithelial neoplasms and are considered as epithelium specific, tumour-associated antibodies.

Approximately 5 μm formalin-fixed tissue sections from 38 primary lung cancers (7 squamous, 5 adenocarcinomas, 4 large cell undifferentiated and 22 small cell carcinomas) ranging from well to poorly differentiated types, were stained with HMFG1 and HMFG2 mabs, using an indirect, two-step immunoperoxidase method.

It has been shown that all the non-small-cell cancers (NSCC) react strongly with the above mabs, regardless of their degree of differentiation. It is concluded, therfore, that these mabs could be a useful tumour marker of NSCC.

On the other hand, the negative reaction of SCC with these mabs could be related to the specific origin of these tumours.

### Immunohistochemical studies of periodontal disease with TAL-IB5 monoclonal antibody

D.V. Skarlos, P. Augustatos, G. Tiniakos, T.A. Adams & A.A. Epenetos

*Pathology Department, NIMTS Hospital, Athens, Greece, Imperial Cancer Research Fund, London, and Royal Postgraduate Medical School and Hammersmith Hospital, London, UK*

TAL-IB5 is a monoclonal antibody which stains all cell types known to express HLA-DR. This antibody works well on formalin-fixed tissue sections.

Using an indirect immunoperoxidase method on frozen or formalin-fixed tissue sections from 24 patients with periodontal disease, it has been shown that TAL-IB5 monoclonal antibody did not stain the lymphocytes which infiltrated the gingivae. Therefore, this indicates that lymphocytes of the gingivae in periodontal disease are not of B-cell origin. Furthermore, using monoclonal antibodies specific for T-lymphocytes, we showed that periodontal disease lymphocytes bear T-suppressor markers (OKT-8, ORTHO). This could be related to an attempt of the host to eliminate the immunological process.

### Blockade of the galactose-binding sites of ricin by photoaffinity-labelling

H.-J. Thiesen[1], M. Brenken[2], L. Hanke[2], H. Paulsen[2] & R. Arndt[1]

[1]*Urologic University Clinic, and* [2]*Institute of Organic Chemistry and Biochemistry, University of Hamburg, FRG*

The cytotoxic action of A-chain conjugates is accelerated or enhanced in the presence of free B-chain or antibody B-chain conjugates. For *in vivo* use, the galactose-binding sites of ricin B-chain have to be blocked in order to diminish their binding to galactose residues on non-target cells.

p-Azidophenyl-$\beta$-D-Galactopyranoside and p-Azidophenyl-$\beta$-D-Lactoside were synthesised and added to ricin recently repurified by Bio-Gel A chromatography. Photolysis was carried out at 254 nm. After modification, the low molecular reactants were separated by ultrafiltration. Modified ricin could be separated from unreacted ricin by Bio-Gel A chromatography. The elution pattern revealed a slightly retarded motion of modified ricin down the column. Efficiencies of inhibition of the ricin fractions were compared in cell assays in the presence of lactose. The results show that the photoaffinity-labelling significantly reduces the non-specific binding to galactose residues. The modified ricin show similar toxicities as unreacted ricin in the presence of lactose (100 mM).

If the degradation of ricin due to photolysis at 254 nm can be reduced by introducing nitrogroups into the phenyl-ring which photolyse at higher wavelengths, higher yields of modified ricin should be obtained.

In the future, the best approach to obtain ricin lacking galactose binding sites would be the recombinant DNA technique of site-directed mutagenesis.

### Removal of B-lymphoma cells from human bone marrow using abrin immunotoxins

Ø. Fodstad, G. Kvalheim, A. Godal, S. Funderud & A. Pihl

*The Norwegian Institute for Cancer Research, Montebello, Oslo, Norway*

Autologous bone marrow transplantation following supralethal treatment with chemotherapy and irradiation is a new approach to the management of B-cell lymphomas. The high frequency of bone marrow infiltration with lymphoma cells has promoted attempts in several laboratories to selectively eliminate the lymphoma cells by treatment of the bone marrow *ex vivo*, by using procedures involving monoclonal antibodies.

Here we report on the use of 2 different immunotoxins for purging bone marrow of lymphoma cells. Whole abrin was conjugated to the antibodies AB3 (anti-HLA DR) and EO1 (anti-common leukocyte antigen). Normal human bone marrow was mixed 1:1 with each of 4 different B-lymphoma cell lines and incubated with the immunotoxins in RPMI medium containing 10% FCS and 0.1 M lactose for 2 h at 37°C. The tumour cell kill, as judged by colony formation in a soft agar assay, was in excess of 2 logs at immunotoxin concentrations (100 ng $ml^{-1}$) that did not affect the clonogenicity of the bone marrow progenitor cells in GM and GEMM assays. Addition of monensin enhanced strongly the tumour cell kill without enhancing the effect on the normal bone marrow cells. Experiments are in progress in which the above procedure is combined with the use of antibody absorbed to monodisperse magnetic polystyrene particles.

### Drug-antibody complexes for immunotherapy

G.A. Pietersz, J. Kanellos, M. Smyth
& I.F.C. McKenzie

*Research Center for Cancer and Transplantation, Department of Pathology, University of Melbourne, Parkville, Australia*

To improve the specificity and efficiency of chemotherapeutic agents, three different drugs (Adriamycin – Ad; Methotrexate – MTX; and Chlorambucil – CBL) were covalently coupled to monoclonal antibodies with specificity for cell surface antigens. The coupling procedures were carefully monitored, and optimised for maximum recovery of protein, antibody and drug activity. The results fell into three different groups: (a) Adriamycin coupled (with difficulty) by the iodacetyl derivative produced conjugates with 8–10 Ad molecules per antibody molecule. On testing *in vitro* and *in vivo* the conjugates were highly specific and active, but considerably less toxic than free adriamycin (although more specific); (b) MTX, activated by N-hydroxysuccinimide, led to conjugates containing 13–15 mol of MTX per antibody molecule. Again, these were highly specific and acted both *in vitro* and *in vivo;* (c) CBL coupled using the same method as for MTX, led to 35 residues of CBL per monoclonal antibody molecule, with good retention of both drug and antibody activity. The CBL conjugates were, again, highly specific, but in addition were at least 6 times more toxic than free CBL. This is one of the first examples where antibody-drug conjugates are *more* active than free drug – and this conjugate could eradicate tumour cells grown in ascites form in mice. Thus carefull attention to drug coupling procedures to give high drug/antibody ratios can lead to production of toxic and specific agents for targeting.

### *In vitro* treatment of colorectal cancer cells with anti-p21

N.A. Habib, H.L. Niman, A. Thompson,
B. Fermor, M.O. Symes, R.C.N. Williamson
& C.B. Wood

*Department of Surgery, Royal Postgraduate Medical School, London, Department of Surgery, Bristol Royal Infirmary, UK and Department of Molecular Biology, Scripps Institute, La Jolla, California, USA*

Proto-oncogenes are involved in normal cell growth. During malignant cell transformation they become activated oncogenes and may produce growth factors. C-*ras* oncogene situated on chromosome 12 is specifically expressed in colorectal carcinomas. To test their potential therapeutic role, monoclonal antibodies against oncogene protein products were applied to human cancer cells obtained from 3 fresh colonic specimens and maintained in cell culture. The antibodies used were developed against specific amino acid sequences of c-*ras* oncogene. Colonic cancer cells were exposed to the antibodies for 24 h and labelled with radioactive selenomethionine for a further 24 h to determine RNA synthesis.

It was found that anti-*ras* monoclonal antibodies caused marked inhibition of RNA synthesis by cultured human cells obtained from all 3 patients with colorectal cancer. The percentages of isotope uptake in treated cells were 60% (73/129 c.p.s.), 24% (138/569) and 17% (25/150) of the uptake by untreated cultured cells (control). By contrast monoclonal antibodies against epidermal growth factor caused no such inhibition, thus confirming the specific action of *ras*-oncogene antibodies on colonic carcinoma cells. The widespread expression of oncogenes and their encoded proteins in human malignancies could provide appropriate therapeutic targets.

### High efficiency antibody iodinations with N-bromosuccinimide

S.J. Mather

*Imperial Cancer Research Fund, London, UK*

The oxidising agent N-bromosuccinimide (NBS) has been investigated as a potential iodinating agent for monoclonal antibodies. A variety of antibodies and antibody fragments have been iodinated with isotopes $^{125}I$ and $^{123}I$. By tailoring the amount of NBS to the amount of iodine used, labelling efficiencies of the order of 95% have been obtained using small quantities of NBS.

Immunoreactivity of labelled antibody (HMFG2) has been tested by ELISA after prolonged exposure (3 h) to NBS without any observable loss of antigen binding.

The achievement of such labelling efficiencies may eliminate the need for subsequent purification procedures. This will be particularly useful in high activity iodinations for antibody-guided radiotherapy.

**Choice of chelate for linking radionuclides or stable nuclides to monoclonal antibodies**

S. Dass, B. Arvind, G.K. Chaturvedi & D.K. Hazra

*Department of Chemistry, DEI Dayalbagh, Agra, and S.N. Medical College, Agra, India*

A variety of agents have been suggested for linking radionuclides to immunoglobulins for radioimmunodetection (RID) and possibly radioimmunotherapy (RIT) and this can be extended to stable elements such as Gadolinium (Gd) for Nuclear Magnetic Resonance (NMR), Boron for neutron activation, and Europium for *in vitro* fluorescence assays. These agents include derivatives of EDTA, DTPA, Deferoxamine, CEDTS, Dextrans and albumin benzoquinones, etc. Of these, five agents have been evaluated, viz. bicyclic anhydrides of DTPA and EDTA, monocyclic anhydride of DTPA, carboxycarbonic anhydride of DTPA and Deferoxamine using both $^{99m}Tc$ and $^{113m}In$ as well as stable analogues of $^{99m}Tc$, i.e. Mn and Re. Among these, cyclic anhydride of DTPA was found to be most useful by reasons of its ease of preparation and characterisation. long shelf life, existence of three nitrogen atoms for efficient linkage to metals and rapid quantitative linkage to immunoglobulins without alteration in their biological behaviour. The other attractive agent was Deferoxamine which is easily available and does not abstract $Ca^{+2}$ and $Mg^{+2}$ ions present in the biological milieu. The problems posed by competing metal ions during the synthesis are emphasised.

**Selection of radionuclide for radioimmunotargeting: An Indian perspective**

D.K. Hazra, S. Dass, A.K. Shukla, S. Saran, M. Kumari, R.N.L. Srivastava, V.L. Lahiri & R. Singh

*S.N. Medical College, Agra, India*

Monoclonal antibodies can be tagged with a variety of radionuclides for radioimmunodetection (RID) or radioimmunotherapy (RIT) either directly or through bifunctional chelating agents. The requirements for RID (pure gamma emission of energy suitable for gamma camera imaging or positron emitting for double photon imaging) are distinct from those for RIT (high LET radiation – alpha, energetic beta or Auger electrons). Further, the radioisotope should have a shelf life permitting visualisation or therapy over the biological time course of accumulation of monoclonal antibody in the tumour, i.e. one to three days, and its daughter products should not be undesirable. Temporal subtraction of early from late images or dual isotope subtraction of non-specific accumulation to reveal specific localisation is possible in RID but not in RIT. In India the non-availability of cyclotron produced radionuclides and the limited neutron flux in the existing reactors impose constraints on the choice of radionuclides, $^{123}I$ and $^{111}In$ being obviously ruled out. This has led us to examine a large number of candidate radionuclides both for RIT and RID considering also the half-life of the radionuclide or its generator parent so as to permit transport from Bombay to our centre and adequate *in vivo* follow-up thereafter. These will now be experimentally evaluated.

**Human tumour xenografts in immunosuppressed mice for evaluating monoclonals**

S. Saran, M.C. Agarwal, B.R. Elhence, K. Singh, I.P. Elhence, S. Khandelwal, R.N.L. Srivastava, M. Kumari, S. Dass, D.K. Hazara & V. Lahiri

*S.N. Medical College, Agra, India*

Human tumour xenografts possess distinct advantages as compared to chemical/viral induced or spontaneous animal tumours for evaluating monoclonal antibodies for clinical use. Nude mice widely employed for such studies are difficult to maintain and also provide an extremely artificial environment for the tumour xenografts. We have used a variety of immunosuppression regimes (combination of irradiation, corticosteroids, cyclophosphamide and cytosine arabinoside) to achieve successful xenografts from human breast carcinomas as well as thyroid carcinomas and Ewing's sarcoma, both directly and after preliminary culture *in vitro*. The histology and antigenic expression of these xenografts has been compared with that of parent tumours as well as *in vitro* tumour explants using monoclonal antibodies and model scanning experiments have been performed. It appears that they can conveniently be used to choose appropriate antibodies for clinical use in radioimmunodetection (RID) or radioimmunotherapy (RIT).

**Imaging of adenocarcinoma in rats with $^{125}$I Mab against carcinoma associated ganglioside antigen**

J. Gretarsdottir[1], L. Jakobsson[1], S. Matteson[1], S.B. Homberg[2], Lo Hafstrom[2], L. Lindholm[3], B. Karlsson[4] & O. Nilsson[4]

*Departments of [1]Radiation Physics, [2]Surgery, [3]Medical Microbiology and [4]Neurochemistry, University of Gothenburg, Sweden*

Two monoclonal antibodies raised against human carcinoma associated ganglioside antigens have been used for experimental immunoscintigraphy. The C50 MAb (IgM) reacts with two ganglioside structures, i.e. Le$^a$ active pentaosylceramide (SiLe$^a$) and sialyted lactotetraosylceramide, and the C241 MAb (IgG1) reacts with only SiLe$^a$.

MAb C50 and C241 were iodinated ($^{125}$I) using the Iodobead method. Experimental colonic carcinoma containing SiLe$^a$ antigen was transplanted as cell suspension on 200 g Wistar Fu rats. Different sized tumours were located in leg muscles, back subcutaneous tissue or liver. The animals were anaesthetised and injected with 2 $\mu$g MAb i.v., activity 0.2 MBq. Gamma camera images were registered 1–72 h after injection. Blood samples were drawn at different intervals. Plasma components were separated by exclusion chromatograph and radioactivity was measured in the fractions. Tumour and organ activities were measured after the animals were sacrificed.

Biological half-time in the whole body was 14 h for C50 and 100 h for C241 (IgG). C50 tumour/blood ratio was 0.2–0.8 at 48 h, tumour/liver ratio was 0.1–1.2 C241 tumour/blood ratio was 0.5–1.4 at 72 h and tumour/liver 1.0–9.8. Column separation of plasma showed at 24 h 88% of $^{125}$I activity bound to MAb, at 48 h 84% and at 72 h 73%. MAb C50 and C241 show tumour/blood ratios that are equal to other MAb used for immunoscintigraphy.

**A comparison of radio-iodinated monoclonal antibody AUA1 with indium-111 radiolabelled AUA1 and iodine-125 labelled F (AB′)$_2$ fragments of AUA1 *in vivo***

D. Snook[1], G. Rowlinson[2], H. Durbin[3], P. Blake[1], F. Brady[2], W.F. Bodmer[3] & A.A. Epenetos[1]

*[1]Royal Postgraduate Medical School and Hammersmith Hospital, London; [2]MRC Cyclotron Unit, Hammersmith Hospital, London and [3]Imperial Cancer Research Fund, London, UK*

The human cervical carcinoma xenograph (HN16) was used as an *in vivo* experimental model to study the immunolocalisation of the monoclonal antibody AUA1. The organ distributions of $^{125}$I-AUA1 and $^{111}$In-AUA1 were compared. $^{111}$In-labelled antibody produced greater tumour to normal organ ratios than $^{125}$I-labelled antibody with the exception of liver and kidney.

In a separate set of experiments, $^{125}$I-labelled F(ab′)$_2$ fragments of AUA1 when compared with whole AUA1 produced better tumour to normal organ ratios due to the rapid blood clearance of the fragments.

F(ab′)$_2$ fragments of monoclonal antibodies offer significant promise for tumour imaging and possible therapy.

**Indium $^{111}$In labelled monocolonal antibody to placental alkaline phosphatase is of clinical value in the detection of neoplasms of testis, ovary and cervix**

A.A Epenetos[1], T. Pawlikowska[1], D. Snook[1], G. Hooker[1], R. Begent[2], H. Durbin[3], R.D.T. Oliver[4], W.F. Bodmer[3] & J.P. Lavender[1]

*[1]Royal Postgraduate Medical School and Hammersmith Hospital, London; [2]Charing Cross Hospital, London; [3]Imperial Cancer Research Fund, London, and [4]Institute of Urology, London, UK*

Indium-111 labelled monoclonal antibody (H17E2) against placental type alkaline phosphatase (PLAP) and testicular placental-like alkaline phosphatase was used in a prospective study of radioimmunoscintigraphy of 15 patients known or suspected to have germ cell carcinoma of testis or carcinoma of ovary or cervix. Good quality images of neoplastic lesions were obtained in the majority of patients with active disease. In two patients with normal conventional radiology, antibody guided imaging located the site of microscopic disease, thus aiding surgical lymphadenectomy. No false positive localisation was seen in patients with PLAP-negative tumours or sites of inflammation such as lung abscess.

This method appears to be a potentially important new adjunct in the diagnosis, staging and monitoring of disease status in patients with PLAP-positive neoplasms such as testicular, ovarian and cervical cancer.

**Placental-type alkaline phosphatase detection by monoclonal antibody (H317): Application in ovarian cancer recurrence**

P.J. McLaughlin[1], M. Critchley[2], P.M. Tromans[3], I.W. McDicken[4] & P.M. Johnson[1]

*Departments of [1]Immunology; [2]Nuclear Medicine; [3]Obstetrics & Gynaecology and [4]Pathology, University of Liverpool, Liverpool, UK*

Placental-type alkaline phosphatase (PLAP) is an oncodevelopmental marker often found in ovarian tumours of epithelial origin. In the present study, a murine IgG1 monoclonal antibody (H317) has been used to measure circulating PLAP in plasma and tumour tissue extracts using a specific enzyme (McLaughlin *et al.*, 1983, *Clin. Chim. Acta*, **130**, 199) and also in fixed tissue sections using H317 in a peroxidase-anti-peroxidase staining technique (McDicken *et al.*, 1983, *Int. J. Cancer*, **32**, 205). This monoclonal antibody was also used in antibody-guided *in vivo* radionuclide imaging in patients after removal of a primary tumour. Of 18 patients suspected of ovarian secondaries, 11 showed focal increased abdominal uptake of the radiolabelled monoclonal antibody of whom 2 also showed diffuse increased abdominal uptake. There was a large 'cold' lesion visible in one patient, confirmed at survey as a cystic tumour. There were 6 normal scans. Unsuspected tumour recurrence was identified in 3 of the 8 clinically clear patients. Detectable plasma PLAP was found in 6 of 13 patients, and 5 of the 6 available tumour tissue extracts contained PLAP. PLAP was demonstrated in 5 of 11 fixed tumour tissue sections by immunohistology.

The radioimmunolocalisation technique using H317 usefully detected tumour recurrence in some ovarian cancer patients. Although plasma PLAP was only detected in 46% of patients, its presence is known to be highly indicative of a tumour as no healthy non-pregnant individuals express the circulative H317-reactive form of PLAP. This is the case even in cigarette smokers (McLaughlin *et al.*, 1984, *J. Clin. Pathol.*, **37**, 826).

**Primary hepatocellular carcinoma localisation using a radiolabelled monoclonal antibody**

N.I. Markham

*Ludwig Institute for Cancer Research, Cambridge, UK*

A rat monoclonal antibody, YPC2/38.8, was selected from a panel of antibodies derived by immunising rats with fresh human colorectal carcinoma. It was found to bind to a 30,000 dalton protein present on the cell surface of normal colon and liver. This protein was increased ten-fold on primary hepatocellular carcinoma (PHC) cells. After labelling with $^{131}I$, YPC2/38.8 was shown to localise human PHCs grown as xenografts in immunosuppressed mice. Eighteen patients with known or suspected PHC were given 1 mg of purified antibody labelled with 1 mCi of $^{131}I$ by slow i.v. injection. In seven out of eight patients with PHC arising in non-cirrhotic livers, good tumour images were obtained on gamma camera or rectilinear scans. In seven patients who had developed PHC on the background of established hepatic cirrhosis, no tumour images were seen. Three patients showed no localisation but diagnoses other than PHC were eventually established. Subsequent studies revealed that the 30K antigen recognised by this antibody was present in increased quantity on PHC cells and the regenerating liver cells in cirrhosis. We conclude that YPC2/38.8 may have potential for the selective targeting of drugs or toxins in patients with PHC arising in a liver unaffected by significant parenchymal disease.

**Detection of unknown metastases using $^{99m}Tc$ or $^{111}In$ labelled F(ab')$^2$ fragments of a moab in 76 patients with melanoma**

G. Paganelli[1,2], P. Riva[1,2], G. Cacciaguerra[1], G. Landi[1], V. Tison[1], M. Marangolo[1] & A. Amadori[1]

*[1]Istituto Oncologico Romagnolo, Italy and [2]Servizio di Medicina Nucleare, Ospedale M. Bufalini, Cesena, Italy*

Seventy-six patients with stage I–IV malignant melanoma were studied with $^{99m}Tc$ or $^{111}In$-labelled F(ab')$^2$ fragments of MoAb 225.28S (Sorin-Biomedica). Seventy-five per cent of previously detected metastases were detectable by immunoscintigraphy. Immunohistology of biopsied tissues confirmed the specificity of antibody. In 26 out of 76 patients, unsuspected positive findings were seen. These findings were divided into two groups:

(1) metastases detectable with conventional techniques performed at the same time as immunoscintigraphy (63%)

(2) metastases not detectable by other methods but confirmed by surgical investigation or by follow-up 3–8 months after antibody scanning (73%).

There was only one false positive scan, caused by free $^{99m}Tc$ in the gut.

### Localisation of tumours with $^{99m}$Tc-monoclonal antibody complexes

G.A. Pietersz, J. Kanellos, J. Baldas, J. Bonnyman & I.F.C. McKenzie

*Research Centre for Cancer and Transplantation, Department of Pathology, University of Melbourne, Parkville, Victoria, Australia*

Although technetium-99m has ideal physical properties for imaging, little use has been made of the radionuclide for labelling monoclonal antibodies – presumably because of labelling difficulties. However, recently a new method for preparing $^{99m}$Tc radiopharmaceuticals based on substitution reactions of $^{99m}TcNCl_4$ has been described. This agent can be prepared in the dry form without the presence of any contaminating metal ions and leads to the formations of Tc chelates in the presence of a Tc nitrido group. Taking advantage of the unique properties of this compound, we decribe a simple method of labelling $\mu$g quantities of monoclonal antibodies for evaluation of the clinical usefulness of $^{99m}$TcN-Mab complexes as diagnostic reagents.

The stability and activity of these complexes were examined *in vitro* with a simple binding assay wich involved antigen positive and antigen negative cells. The complexes were shown to be highly specific and would bind antigen positive cells 10 times greater than antigen negative cells.

In an *in vivo* experimental model $(BL6 \times BALB/c)F_1$ mice carrying palpable ITT(1) 75NS tumours (0.4–1.5 cm diameter) were injected i.v. via tail vein with either of two monoclonal antibodies labelled with $^{99m}TcNCl_4$. Mice were either scanned with a gamma camera or their tissues were removed and the localisation of radiolabelled antibody calculated as cpm $g^{-1}$ of organ, results were calculated as a ratio of the tissue to blood distribution. The studies showed that specific localisation had occurred. Tumours (4 mm) could be successfully imaged *without* the need for background subtraction.

### Treatment of advanced B-cell lymphoma with monoclonal anti-idiotype antibodies

A. Hekman, A. Honselaar & E.M. Rankin

*Division of Immunology, The Netherlands Cancer Institute, Amsterdam, The Netherlands*

Four patients with non-Hodgkin's lymphoma have been treated with mouse monoclonal antibodies (moab) directed against the idiotype of the tumour immunoglobin. Two moabs were IgG2a and two IgG1. Single dose of 10 mg to 1200 mg were infused over 4 to 48 h in an escalating schedule reaching total amounts of 3.8 to 4.8 g over periods of 3 weeks to 2 months. Patients were monitored for binding of moab to malignant cells in different sites, serum moab level, level of serum idiotypic Ig if present, clinical response, side effects and anti-mouse antibodies. Infusion of anti-idiotype resulted in transient falls in circulating lymphocyte counts and/or temporary removal of circulating idiotype. Binding to peripheral tumour cells was seen before complete clearance of free idiotype. After doses exceeding saturation of cells and removal of free idiotype, free moab was detected in the serum of 3 patients, but not in the fourth. Binding of moab *in vivo* to tumour cells in lymph nodes, bone marrow and ascites could be demonstrated. Overall therapeutic effect was minimal, 3 patients showed a slight, ~10%, decrease in lymph node size, in 1 patient the tumour was unaffected. There was no evidence of modulation in 2 patients. In one case the circulating tumour cells showed a reversible decrease in antigen density, but never became idiotype negative. One patient showed an abrupt increase in modulation *in vivo* after 2 weeks of treatment. Experiments *in vitro* showed that this was due to the removal of a discrete subpopulation of smaller, nonmodulating tumour cells. Apart from one episode of acute hypotension, side effects were generally minimal; there was no decrease of kidney or liver function. None of the patients made anti-mouse antibodies.